# Coarse-Grained Simulations of Crystallization in Phase-Separated Polymer Blends with Block Copolymer Compatibilizers

**DOI:** 10.1021/acs.macromol.5c01767

**Published:** 2025-11-21

**Authors:** Yunjia Zhang, Wenlin Zhang

**Affiliations:** Department of Chemistry, 3728 Dartmouth College, 41 College St., Hanover 03755, New Hampshire, United States

## Abstract

We employ coarse-grained molecular dynamics simulations to investigate crystallization in binary blends of phase-separated semicrystalline polymers with and without block copolymer compatibilizers. To mimic realistic semicrystalline polymer blends, we introduce mismatches in monomer sizes of different polymers to avoid artificial cocrystallization. By tuning the intermolecular interactions, we adjust the incompatibility and the width of the interfaces between different polymers. We find that broad interfaces significantly hinder crystallization and crystal stem growth, not due to entanglement constraints, but because of the presence of incompatible species near the interface. The suppression of crystallization extends beyond the interfacial region. Adding block copolymer compatibilizers, which impede disentanglement near the interface, further reduces interfacial crystallinity. While long copolymers can form tie bridges across interfaces and offer potential mechanical reinforcement, they also hinder the formation of entangled loop bridges by reducing crystallization near interfaces.

## Graphical abstract

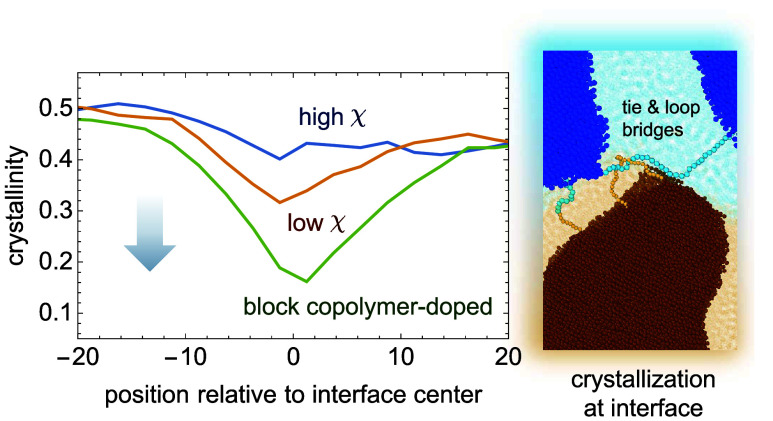

## Introduction

Interfaces are ubiquitous in polymer materials and are central to the performance of many industrial processes and emerging technologies, including additive manufacturing, multilayer film production, and mechanical plastic recycling.^1–4^ These interfaces may either form spontaneously due to the incompatibility between polymer species or be deliberately engineered to achieve specific materials design. Regardless of their origin, interfacial regions are often zones of mechanical weakness. Poor adhesion or phase separation across interfaces results in reduced fracture resistance, ultimately limiting the mechanical performance of otherwise high-strength polymer systems. Controlling the molecular-scale structure and dynamics at polymer interfaces is key to materials design and processing.

For semicrystalline polymer blends, the material properties are also governed by crystallization and the resulting semicrystalline morphologies near the interfaces.^5,6^ However, the effects of phase-separated polymer interfaces on crystallization are not well understood. Some studies reported enhanced crystallization near interfaces,^7–10^ possibly due to heterogeneous nucleation induced by interfaces.^11^ Others have found that crystallization is suppressed at interfaces, with crystalline domains preferentially growing in the bulk regions.^12,13^ These discrepancies likely arise from a multitude of contributing factors—including polymer compatibility,^14^ composition,^13,15^ and flow/processing history,^4,16–18^ which complicate efforts to predict or control semicrystalline morphology near interfaces.

Compared to experiments, simulations can exclude competing effects and provide microscopic insight into polymer crystallization behaviors near various interfaces. Although a few studies have explored crystallization at polymer blend interfaces, they typically involve one crystallizable species and one amorphous or noncrystallizable component.^10,12,14^ Prior simulation studies also revealed how polymers crystallize near impenetrable surfaces and substrates.^19–22^ Nonetheless, direct simulations of crystallization in phase-separated domains of two semicrystalline polymers and near their interfaces are still mostly lacking.

To improve interfacial adhesion and mechanical performance in semicrystalline polymer blends, block copolymer compatibilizers have been employed. These molecules, with architectures such as diblock,^23,24^ multiblock,^25–27^ and graft copolymers,^28^ reduce interfacial tension and form bridge structures by anchoring each block into its preferred homopolymer phase. Well-designed block copolymers can even crystallize in different polymer domains and act as tie bridges, transmitting stress across otherwise weak interfaces. While the thermodynamic and mechanical effects of compatibilizers have been studied, their impact on interfacial crystallization remains underexplored at the microscopic level. Notably, some experimental reports have observed a reduction in crystallinity near interfaces upon the addition of compatibilizers,^26,29,30^ although the underlying mechanisms remain poorly understood at the molecular level. Revealing the effects of block copolymer architecture and loading on local crystallinity, lamella growth, and entanglement topology near interfaces could help optimize the semicrystalline polymer interfaces.

In this work, we employ coarse-grained molecular dynamics simulations to systematically investigate crystallization at immiscible polymer interfaces, with and without block copolymer compatibilizers. Our simulations reveal that broad interfaces between weakly incompatible polymers significantly suppress crystallization and limit lamellar thickening, not due to entanglement constraints but instead results from compositional disorder. The suppression extends well beyond the width of the compositional interface. Sharper interfaces between highly incompatible polymers, however, impose moderate hindrance on crystallization near interfaces. Because crystals prefer to nucleate away from interfaces with random orientation, the crystalline stems force chains in the interfacial region to align parallel to the stem when growing near the interfaces and thus distort and roughen the interfaces.

The addition of block copolymers can further hinder crystallization near interfaces. We show that the block junctions of these compatibilizers are confined to the interfacial regions. The middle sectors of the block copolymers relax slowly and in turn hinder the crystallization in the interfacial regions. By applying topological analyses to the semicrystalline interfaces, we also quantify the formation of loop bridges and tie bridges connecting different crystalline domains across the interfaces. Overall, our findings provide mechanistic insight into the suppression of crystallization by block copolymer compatibilizers. We show that these block copolymers act as both structural reinforcers and crystallization inhibitors. We expect our work to help guide the rational design of semicrystalline polymer blends with optimized interfacial properties.

## Methods

In this study, we perform coarse-grained (CG) simulations using the GROMACS simulation package.^31^ Our CG model is similar to those used in recent studies of crystallization in semicrystalline homopolymers.^32,33^ In our simulations, each polymer chain consists of *N* = 200 coarse-grained beads of type A or B, connected by harmonic springs with a stretching potential

1 $$U_{\mathrm{bond}}=\frac{1}{2}k_{0}{\left(l-l_{\mathrm{ij}}\right)}^{2}$$

where *k*_0_ = 127 *u*/*a* ^2^ is the spring constant, and *l*_*ij*_ denotes the equilibrium bond length between beads of type *i* and *j*. We use reduced units *a* and *u* for length and energy, respectively. Thus, in our simulations, the reduced time $\tau =\sqrt{m_{b}a^{2}/u}$ , where *m*_b_ is the bead mass, which is the same for *A* and *B* beads. The time step for our molecular dynamics simulations is Δ*t* = 5 × 10^–3^ τ. To prevent cocrystallization, we reduce the size of *B* beads to 75% of that of *A* beads, a ratio inspired by the lattice dimensions of polyethylene and isotactic polypropylene crystals.^34,35^ We accordingly scale the bond lengths in homopolymers *A* and *B* and the block copolymers as *l_AA_* = *a*, *l_BB_* = 0.75 *a*, and *l_AB_* = 0.875 *a*.

The nonbonded interactions are described using a truncated and shifted Lennard-Jones (LJ) potential

2 $$U_{\mathrm{ij}}\left(r\right)=\{\begin{array}{ll} 4\epsilon_{\mathrm{ij}}u\left[{\left(\frac{\sigma_{\mathrm{ij}}}{r}\right)}^{12}-{\left(\frac{\sigma_{\mathrm{ij}}}{r}\right)}^{6}\right]-U_{\mathrm{ij}}\left(r_{c}\right) & \mathrm{if}\;r<r_{c}\\ 0 & \mathrm{if}\;r\geq r_{c} \end{array}$$

where *r*_c_ = 4.725 *a* is the cutoff distance, and the potential is shifted by

3 $$U_{\mathrm{ij}}\left(r_{c}\right)=4\epsilon_{\mathrm{ij}}u\left[{\left(\frac{\sigma_{\mathrm{ij}}}{r_{c}}\right)}^{12}-{\left(\frac{\sigma_{\mathrm{ij}}}{r_{c}}\right)}^{6}\right]$$

We set σ_*ij*_ = 1.89 *l*_*ij*_ to enhance lamellar crystal formation.^36^ The rather large bead diameter introduces bending stiffness through 1,3-repulsive interactions, promoting local alignment of bonded beads. Standard bead–spring polymer chains with moderate harmonic bending potentials cannot easily form lamellar crystals of folded chains.^37,38^

To control polymer compatibility, we vary the depth of the LJ potential well, ϵ_*ij*_. We set ϵ_*AA*_ = ϵ_*BB*_ = 1 and tune ϵ_*AB*_ to adjust the incompatibility between polymers *A* and *B*. Weakly incompatible blends with ϵ_*AB*_ = 0.99 exhibit broad interfaces, while highly incompatible blends with ϵ_*AB*_ = 0.9 represent sharper interfaces due to a stronger effective *A*–*B* repulsion. By fitting the interfacial profiles using the Helfand-Tagami theory,^39^ we estimate the Flory–Huggins χ for the two molten samples as 0.027 and 0.417 (see Supporting Information for more details).

To identify an appropriate temperature for crystallization simulations, we first estimate the glass transition temperatures (*T*_g_) of homopolymers *A* and *B*. We quench molten homopolymers from *T* = 4 *u*/*k*_*B*_ to a range of lower temperatures, equilibrate for 22.8 kτ under NPT conditions at zero pressure, from which their steady-state densities ρ­(*T*) are recorded. We use a stochastic v-rescale thermostat and a Parrinello–Rahman barostat. As shown in Figure 1a, we determine *T*_g_ from the intersection of linear fits to the high- and low-temperature branches of ρ­(*T*), yielding *T*_g_ = 1.89 *u*/*k*_*B*_ for polymer *A* and 1.78 *u*/*k*_*B*_ for polymer *B*.

**Figure 1:**
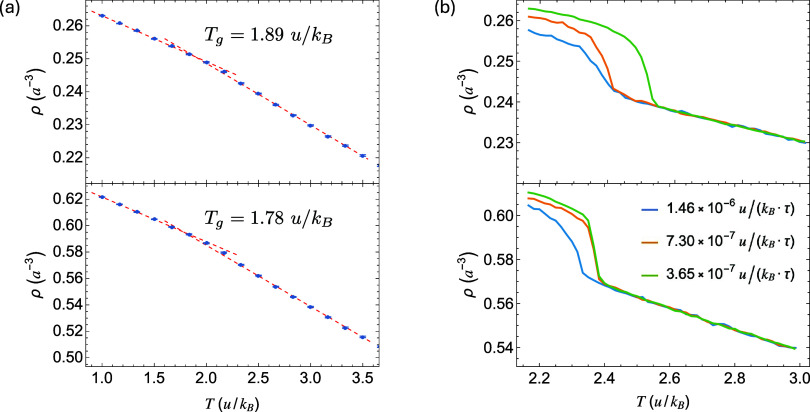
(a) Number density ρ vs temperature *T* for amorphous polymers *A* (top) and *B* (bottom). (b) ρ vs *T* at different cooling rates for polymers *A* (top) and *B* (bottom).

The ideal temperature for simulations of isothermal crystallization should be much higher than *T*_g_ to ensure fast polymer relaxation but sufficiently lower than the equilibrium melting temperature *T*_m_ to provide a strong thermodynamic driving force for the phase transition. Instead of precisely obtaining *T*_m_, we estimate the lower bound of crystal melting temperature by cooling the equilibrated melts from *T* = 3 *u*/*k*_*B*_ to *T* = 2.17 *u*/*k*_*B*_ at different rates (Figure 1b). Sudden density increases indicate crystallization, and the corresponding temperatures define the onset crystallization temperatures *T*_cry_, which are below *T*_m_ and are dependent on the cooling rate. At the slowest cooling rate, *T*_cry_ ≈ 2.54 *u*/*k*_*B*_ for polymer *A* and 2.38 *u*/*k*_*B*_ for polymer *B*. By heating the samples starting from the semicrystalline states (obtained using the slowest quench rate of 3.65 × 10^–7^ *u*/(*k*_*B*_ ·τ)), we observe that the apparent melting temperatures *T*_m_ are significantly higher than the corresponding *T*_cry_, measured as *T*_m_ = 3.1 *u*/*k*_*B*_ for *A* and *T*_m_ = 2.8 *u*/*k*_*B*_ for *B* (Figure S1). This gap between *T*_cry_ and *T*_m_ may arise from the formation of some mesophase during crystallization, as proposed in previous studies.^40^ Identifying the mesophase during the multistep crystallization, however, is beyond the scope of this paper. Based on these results, we choose *T* = 2.33 *u*/*k*_*B*_ for isothermal crystallization simulations—a temperature above both *T*_g_ values yet sufficiently low to induce homogeneous nucleation within practical simulation time scales.

To prepare phase-separated blends with planar interfaces, we first confine a melt of 150 *A* chains or 300 *B* chains between two flat and impenetrable walls perpendicular to *ẑ*. The periodic boundary condition is applied in the *x̂* and *ŷ* directions. We then construct a binary blend with sharp interfaces by gluing the A and B slabs together, removing the walls, and reinstating periodic boundary conditions in *ẑ*. The system is equilibrated at *T* = 3 *u*/*k*_*B*_ for 0.114 Mτ, allowing interdiffusion of the two species across the interface. The pressures along *x̂* and *ŷ* are maintained at zero using the Parrinello–Rahman barostat,^41^ while the box dimension in the *ẑ* direction is kept at 126.7 *a*. During equilibration, the initially sharp interfaces broaden and eventually stabilize. We extend the NPT simulation for the equilibrated blend for an additional 1.369 Mτ and extract 12 independent configurations, separated by more than twice the conformational relaxation time.

We verify the initial configurations for isothermal crystallization are not correlated by computing the end-to-end vector correlation (*C*_*i*,*i*+1_ = [⟨(*R*_*i*_ – *R̅*)·(*R*_*i*+1_ – *R̅*)⟩]/[⟨(*R*_*i*_ – *R̅*)^2^⟩], where *R*_*i*_ represents the end-to-end vector of a polymer chain in the *i*th snapshot, *R̅* is the mean end-to-end displacement, and ⟨⟩ denotes the average over all polymers), which is less than 0.052. After quenching the equilibrated blends to *T* = 2.33 *u*/*k*_*B*_, we crystallize the phase-separated blends for 1.597 Mτ. Unless otherwise stated, all the measurements in Results and Discussion are averaged over 12 crystallization trajectories for each system.

To identify crystalline atoms in simulations, we calculate the local nematic order tensor for a given atom

4 $$Q_{\mathrm{ij}}=\frac{1}{n}\sum_{k=1}^{n}\left(t_{i}^{k}t_{j}^{k}-\frac{1}{3}\delta_{\mathrm{ij}}\right)$$

where *n* is the number of same-type atoms within a 4.45 *a* cutoff from the reference atom, and *t* ^*k*^ is the unit bond vector of neighbor *k*, and indices *i*,*j* = (*x*,*y*,*z*). The scalar order parameter is computed as *S* = 1.5 λ, where λ is the largest eigenvalue of *Q*. The cutoff of 4.45 *a* corresponds to the average location of the second peaks in the radial distribution functions of the two polymers. Atoms with *S* > 0.8 are classified as crystalline. Snapshots of crystallized systems with sharp and broad interfaces are shown in Figure 2.

**Figure 2:**
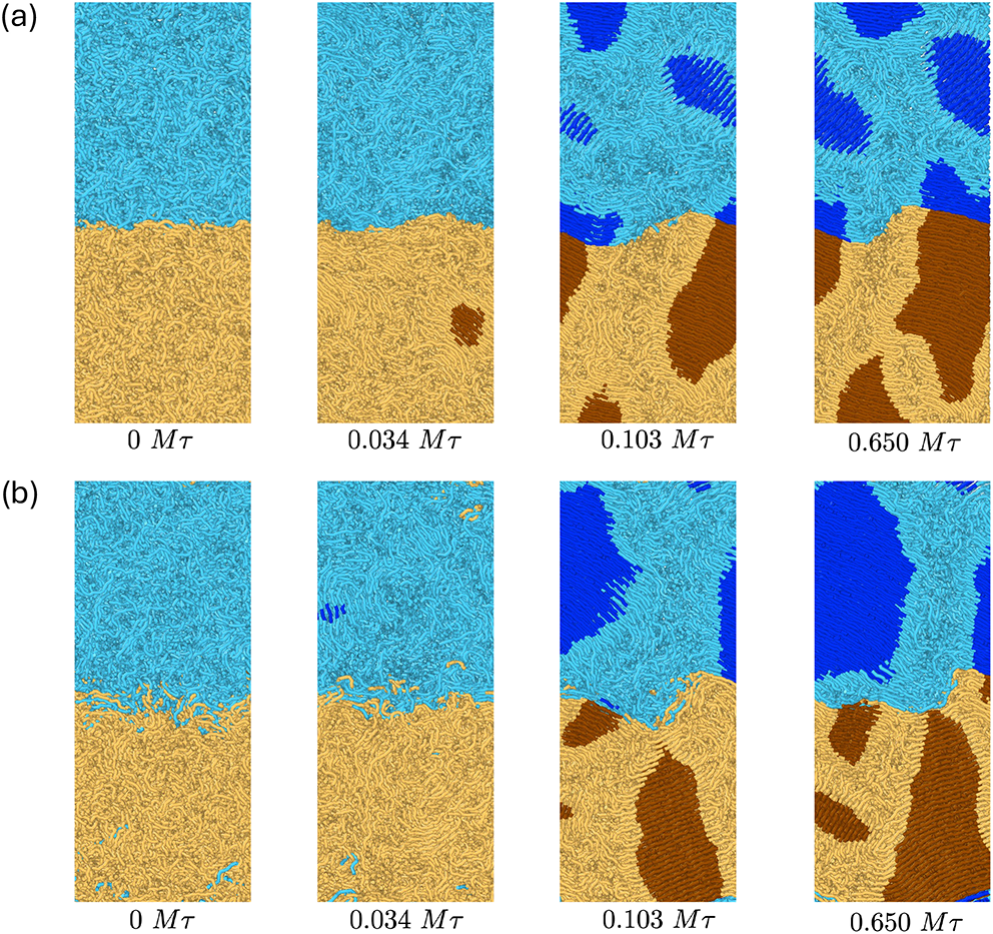
Snapshots of binary blends during crystallization with ϵ_*AB*_ = 0.9 (a) and ϵ_*AB*_ = 0.99 (b). Crystalline atoms (darker colors).

Entanglements play an important role in polymer crystallization. We use the Z1+ algorithm^42^ to identify entanglement kinks in the simulation, including their spatial positions and the pair of polymer chains involved in each kink, from which we compute the local entanglement density ρ_e_. We compare ρ_e_ to the unperturbed entanglement density

5 $$\rho_{e}^{0}=\rho_{e}^{A0}{\mathrm{\rho ̃}}_{A}+\rho_{e}^{B0}{\mathrm{\rho ̃}}_{B}$$

where ρ_e_^*A*0^ and ρ_e_^*B*0^ are the bulk entanglement densities of pure polymers *A* and *B*, measured after cooling but before crystallization, and ρ̃_*i*_ is the local volume fraction of species *i*. By normalizing ρ_e_ with respect to ρ_e_^0^ and the local amorphous fraction, 1 – Φ_c_, where Φ_c_ is the local crystallinity, we obtain the relative local entanglement density

6 $$\tilde{\rho_{e}}=\frac{\rho_{e}}{\left(1-\Phi_{c}\right)\rho_{e}^{0}}$$

We normalize the local entanglement density with respect to the local amorphous fraction because entanglements only form in the amorphous regions.

To assess how block copolymers affect crystallization, we introduce a thin interfacial layer of diblock copolymers between homopolymer *A* and *B* slabs. We vary the overall molecular weight of the diblock copolymers (denoted by *A*_38_ *B*_50_, *A*_75_ *B*_100_, *A*_150_ *B*_200_, and *A*_225_ *B*_300_) while maintaining the same contour lengths for the two blocks. To probe the effects of block copolymer loading, either 12 or 24 diblock copolymers are added to each interface. We adjust the total number of homopolymers to maintain a consistent system size across all simulations. A full summary of blend compositions is in Supporting Information. Following the same equilibration protocols, we create initial configurations of phase-separated blends with interfaces compatibilized by block copolymers. By quenching the blends to *T* = 2.33 *u*/*k*_*B*_, our simulations reveal the effects of block copolymer loading and length on the crystallization and semicrystalline morphologies near the interfaces of phase-separated blends.

## Results and Discussion

### Homopolymer Crystallization near Interfaces

By varying the interspecies interactions, we create two polymer blends with different interfaces but otherwise identical. To quantify the interfaces, we compute the volume fraction profile of each species as ρ̃_*i*_(*d*) = ρ_*i*_(*d*)/ρ_*i*_^0^, where ρ_*i*_(*d*) is the local density of species *i* at a distance *d* from the interface, and ρ_*i*_^0^ is its bulk density. We fit ρ̃_*A*_(*d*) to the Helfand–Tagami theory for asymmetric polymer blends,^39^ yielding interfacial widths of 2.28 *a* for the sharp interface and 9.60 *a* for the broad interface.

We expect the broad interfaces in our simulations—with widths about 4.9 times the statistical segment length of polymer *B*—to mimic the interfaces between common semicrystalline polyolefin blends. For conceptual references, the interfacial width between head-to-head polypropylene (hhPP) and polyethylene (PE) at 450 K, estimated from their Flory–Huggins χ parameter,^43^ is about 7 nm and thus 12 times the statistical segment length of PE *b*_PE_ ≈ 0.59 nm.^44^ The interfaces between phase-separated polyolefins are expected to be about 3–5 nm,^27^ which are about 5 to 8 times *b*_PE_.

Upon crystallization, the initially flat interfaces, aligned parallel to the *xy*-plane, become distorted and narrower (Figure 3a). To characterize the shape of the interface, we discretize the interfacial region into 900 cells. Within each cell, we identify the location for ρ̃_*A*_(*z*) = ρ̃_*B*_(*z*), and construct the interface via linear interpolation. The distance from each atom to the interface is calculated as the shortest distance *d* to the interpolated surface. We assign atoms on the *A*-rich side and *B*-rich side of the interface by *d* > 0 and *d* < 0, respectively. We will use the calculated distance from each atom to the interface to quantify the crystallization behavior of the polymer chains near the interfacial region. The exclusion of impurities from growing crystals near the interfaces, combined with enhanced incompatibility in the melt at lower temperatures, leads to interface sharpening. The interfacial width decreases from 2.28 to 1.68 *a* in samples with sharp interfaces and from 9.60 to 3.43 *a* in weakly incompatible samples (Figures 3b,d and S2).

**Figure 3:**
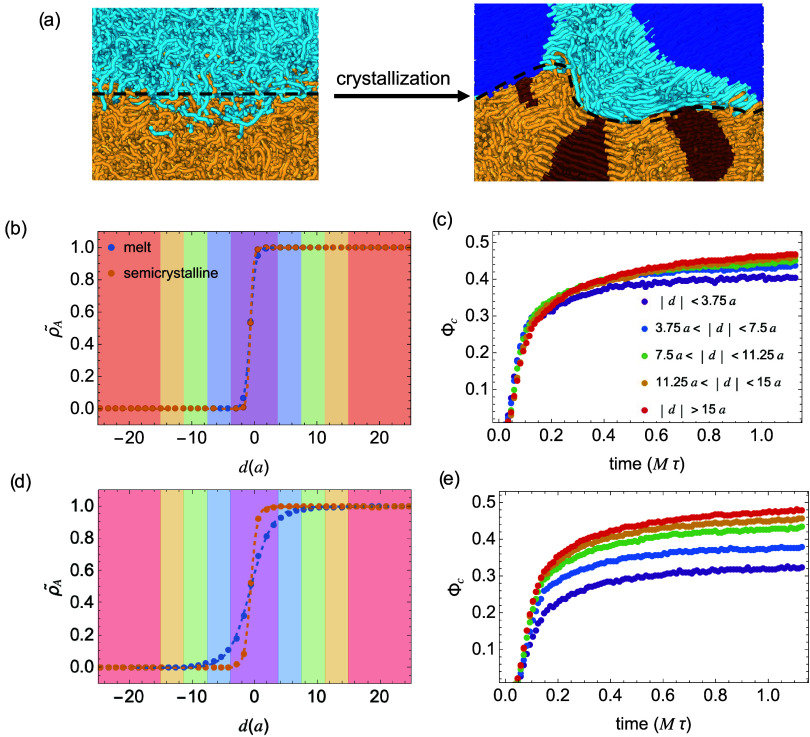
(a) Snapshots of semicrystalline interfaces (ϵ_*AB*_ = 0.99, broad interface) before and after crystallization. Centers of interfaces are marked by dashed curves. (b, d) Volume fraction profiles of polymer *A* ρ̃_*A*_ near sharp (b) and broad (d) interfaces before and after crystallization. (c, e) Local crystallinity Φ_c_ vs time for atoms near (c) sharp interfaces and (e) broad interfaces. Different colors correspond to the shaded regions in (b) and (d).

We notice that the compositional inhomogeneity near the interfaces (Figure 3b,d) hinders the nucleation and growth of the crystal. Figure 3c,e illustrates the time evolution of local crystallinity Φ_c_(|*d*|)—defined as the fraction of crystalline atoms—for systems with sharp and broad interfaces. In systems with sharp interfaces, both the crystallization kinetics and the final crystallinity are comparable across the simulation box. In contrast, crystal growth near broad interfaces is markedly hindered, consistent with what we observed in our previous study on PE crystallization at PE/*i*PP interface.^12^ This effect is especially evident during the early stage of crystallization, as indicated by the slope of the crystallinity curve before 0.2 Mτ, which decreases for atoms located closer to the interface. In addition to slow crystallization kinetics and reduced final crystallinity, the lamellar thickness near broad interfaces is also reduced. As shown in Figure 4, the average crystal stem lengths near broad interfaces are systematically shorter than those in the bulk.

**Figure 4:**
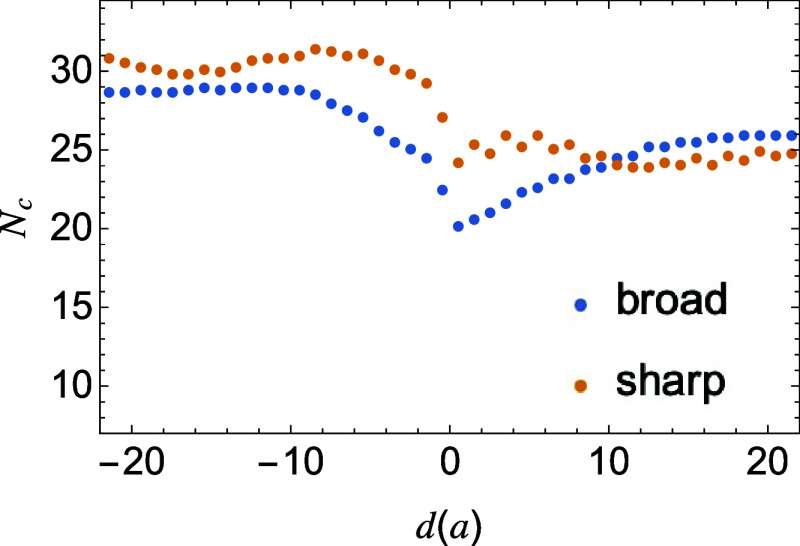
Local crystal stem length *N*_c_ vs distance from the interface for both sharp and broad interfaces.

Although crystals prefer to nucleate outside the interfacial region, crystallization can propagate toward the interface and distort the flat interfaces. To see this, we quantify the interfacial roughening using ⟨*P*_2_(*n̂* ·*ẑ*)⟩, where *ẑ* is the *z*-axis, *n̂* is the local interface normal, *P*_2_ is the second-order Legendre polynomial, and ⟨⟩ denotes average over the interface. Before crystallization, ⟨*P*_2_(*n̂* ·*ẑ*)⟩ is about unity in both polymer blends, representing the flat interfaces. After crystallization for sufficient time, both the broad and narrow interfaces are distorted, indicated by the order parameters less than unity ⟨*P*_2_(*n̂* ·*ẑ*)⟩= 0.63 and 0.82, respectively.

As crystallites with random orientations grow into the interfacial region, the advancing crystalline lamellae impact the local orientation of the interface. Specifically, molten chains tend to align parallel to the rigid crystal surfaces, which changes the local interfacial normal *n̂*. To quantify the local orientation, we compute *P*_2_(*n̂* ·*ĉ*), where *ĉ* is the unit vector along a crystalline stem (Figure 5). The negative *P*_2_(*n̂* ·*ĉ*) indicates that the crystalline stems are parallel to both the sharp and broad interfaces. Thus, in weakly incompatible blends, the randomly oriented stems, propagated from the bulk regions, distort the flat interfaces and increase interfacial roughness. In highly incompatible polymer blends, crystalline nuclei have a higher probability of formation close to the center of the narrow compositional interface and exhibit orientations roughly parallel to the interface. Thus, the propagation of these crystalline stems only moderately distorts the flat interfaces.

**Figure 5:**
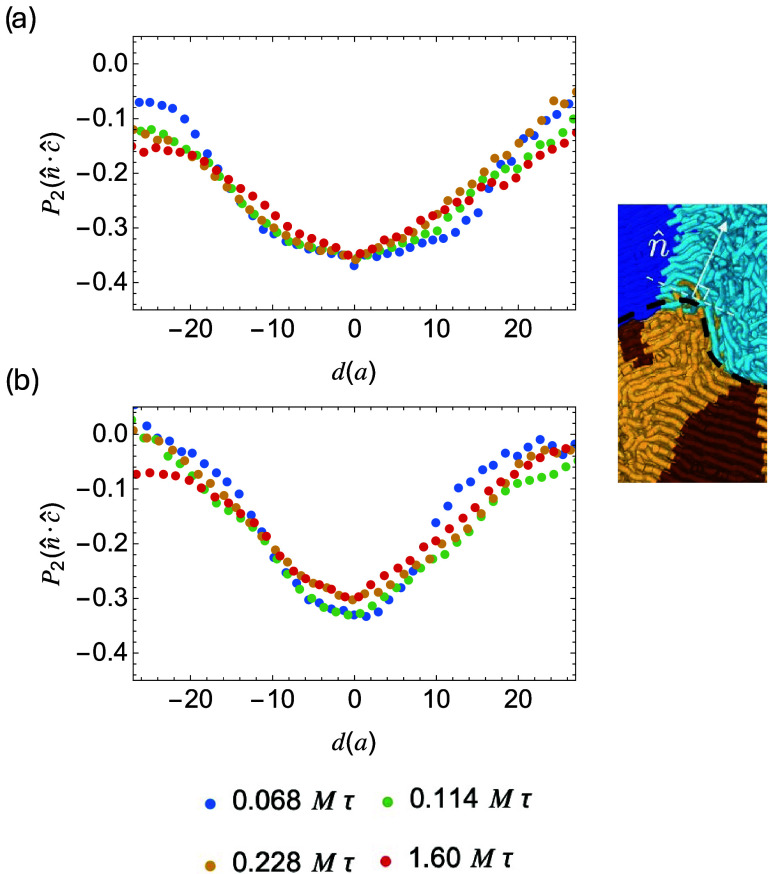
Local order *P*_2_(*n̂* ·*ĉ*) of crystalline stems with orientation *ĉ* and interface normal *n̂* for broad (a) and sharp (b) interfaces during crystallization.

To evaluate the entanglement density at interfaces, we measure the distance of each entanglement point to the interface and compute the local entanglement density as a function of distance, following the procedure described in the Methods section. We find that the entanglement density remains lower near the interface compared to the bulk (Figure 6). This reduction in entanglement is a result of phase separation between incompatible polymer species, as shown in previous works.^45,46^

**Figure 6:**
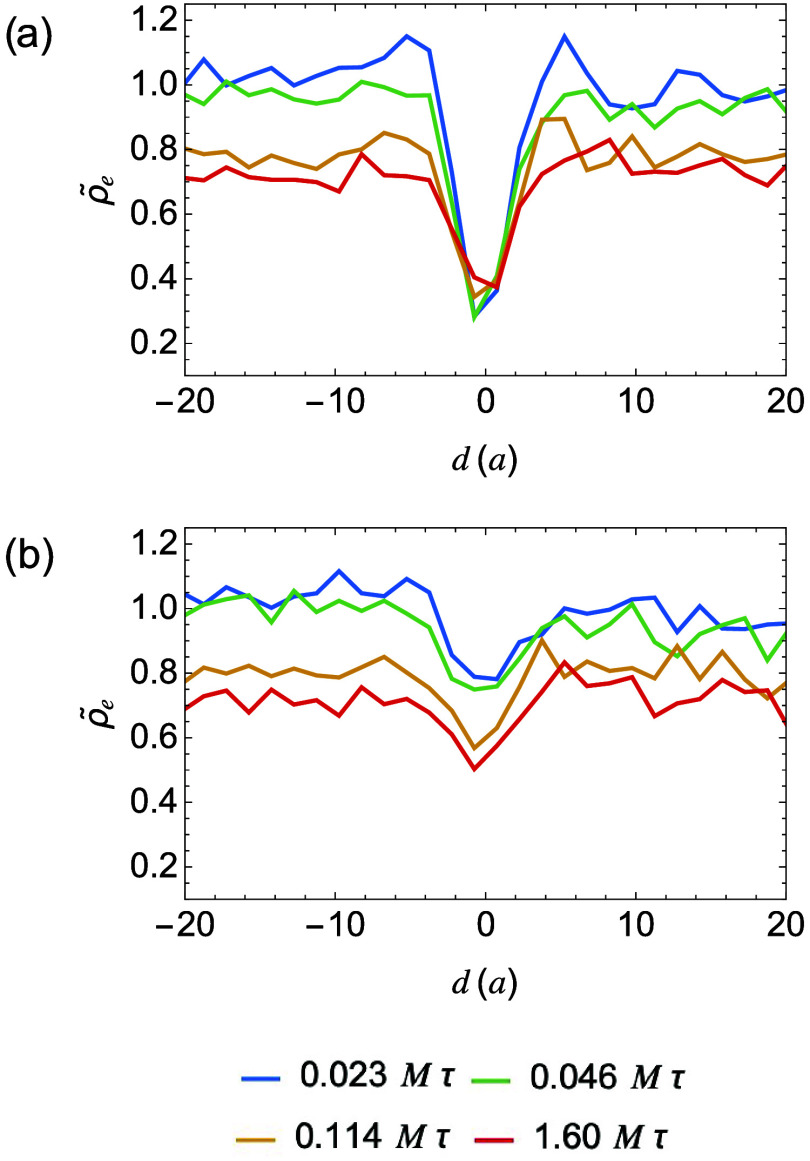
Normalized amorphous entanglement density profiles, ρ̃_*e*_ vs distance from the interface for sharp (a) and broad (b) interfaces of homopolymers at different crystallization times.

Several mechanisms may account for the decreased crystallinity and reduced stem length at broad interfaces. Although prior studies suggest that topologically trapped entanglement kinks can restrict stem growth,^47,48^ the observed lower interfacial entanglement density here cannot explain the suppression; if anything, fewer constraints would ease growth. Instead, comparison of sharp and broad interfaces indicates that compositional impurities at the interface dominate: because the two polymers are incompatible in the crystalline phase, chains from the other species act as impurities that must be excluded from the crystalline nuclei. Thus, the translational entropy penalty for demixing lowers the thermodynamic driving force for crystallization, in turn increases the nucleation barrier and hinders the crystallization kinetics. A more detailed discussion on the effects of compositional inhomogeneity on polymer crystallization can be found in a previous work.^14^

Although the suppressed crystallization arises from the impurities in the interfacial region, the blends exhibit reduced crystallinity and lamellar thickness over wide regions across the interfaces. While the final width of the blend interface is ∼3.43 *a*, the zone of reduced crystallinity and shorter stems spans nearly 15 *a*. We expect that the rather long-ranged crystallization reduction arises from the interfacial free energy penalty for abrupt changes in stem length within a crystalline lamellae. Even outside the crystalline domains, the crystalline order and stem length decay smoothly over the thickness of a mesomorphic phase until reaching the disordered molten phase.^40^

To see this, we derive the form of the normalized stem length distribution *Ñ*_c_(*r*) = (*N*_c_(*r*) – *N̅*_c_)/*N̅*_c_, where *N*_c_(*r*) is the local stem length and *N̅*_c_ is the bulk average, by minimizing a phenomenological Landau–Ginzburg free energy in which a square-gradient term penalizes the stem-length variations within a crystallite (see Supporting Information)­

7 $$\tilde{N_{c}}\left(r\right)=\lambda_{i}\left[\tanh^{2}\left(\frac{r}{\xi_{i}}\right)-1\right]$$

Here, λ_*i*_ and ξ_*i*_ are fitting parameters. λ_*i*_ characterizes the degree of relative stem-growth suppression near the interface, while ξ_*i*_ represents the spatial correlation length of *Ñ*_c_(*r*). The functional form provides an excellent description of the simulated stem-length profiles (Figure 7). The values of λ_*i*_ are comparable for the two polymers (0.23 for *A* and 0.29 for *B*), consistent with their similar molecular architectures. In contrast, ξ_*i*_ differs more significantly: ξ_*A*_ ≈ 9.0 *d*_*A*_ and ξ_*B*_ ≈ 7.8 *d*_*B*_. Polymer *A* with a higher melting temperature exhibits a longer correlation length of lamellar thickness near the interface.

**Figure 7:**
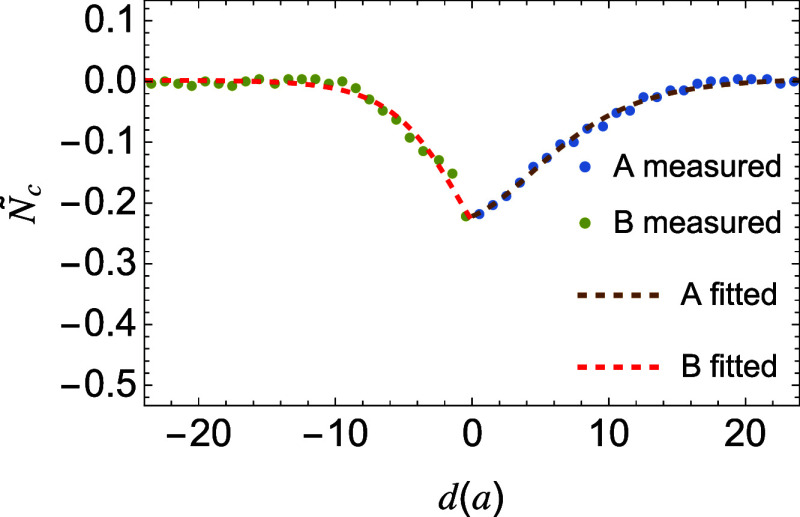
Normalized crystal stem length *Ñ*_*c*_ profile in the broad interface. Fits using eq 7 (curves).

### Block Copolymers at Semicrystalline Polymer Interfaces

To investigate how block copolymers affect crystallization near polymer–polymer interfaces, we systematically introduce symmetric diblock copolymers of varying lengths and loadings into blends with either sharp or broad interfaces, as described in the 2Methods section. In the equilibrated melt, the block copolymer junctions preferentially localize near the interface, and the chain ends extend into their preferred phases. This distribution is confirmed by both visual snapshots (Figure 8a) and analysis of the average absolute distance to the interface, |*d*|, for each bead in the copolymer (Figure 8b). As expected, longer block copolymers penetrate more deeply into the bulk regions. The distributions of junctions in broad interfaces are wider, indicating weaker confinement at the interfaces.

**Figure 8:**
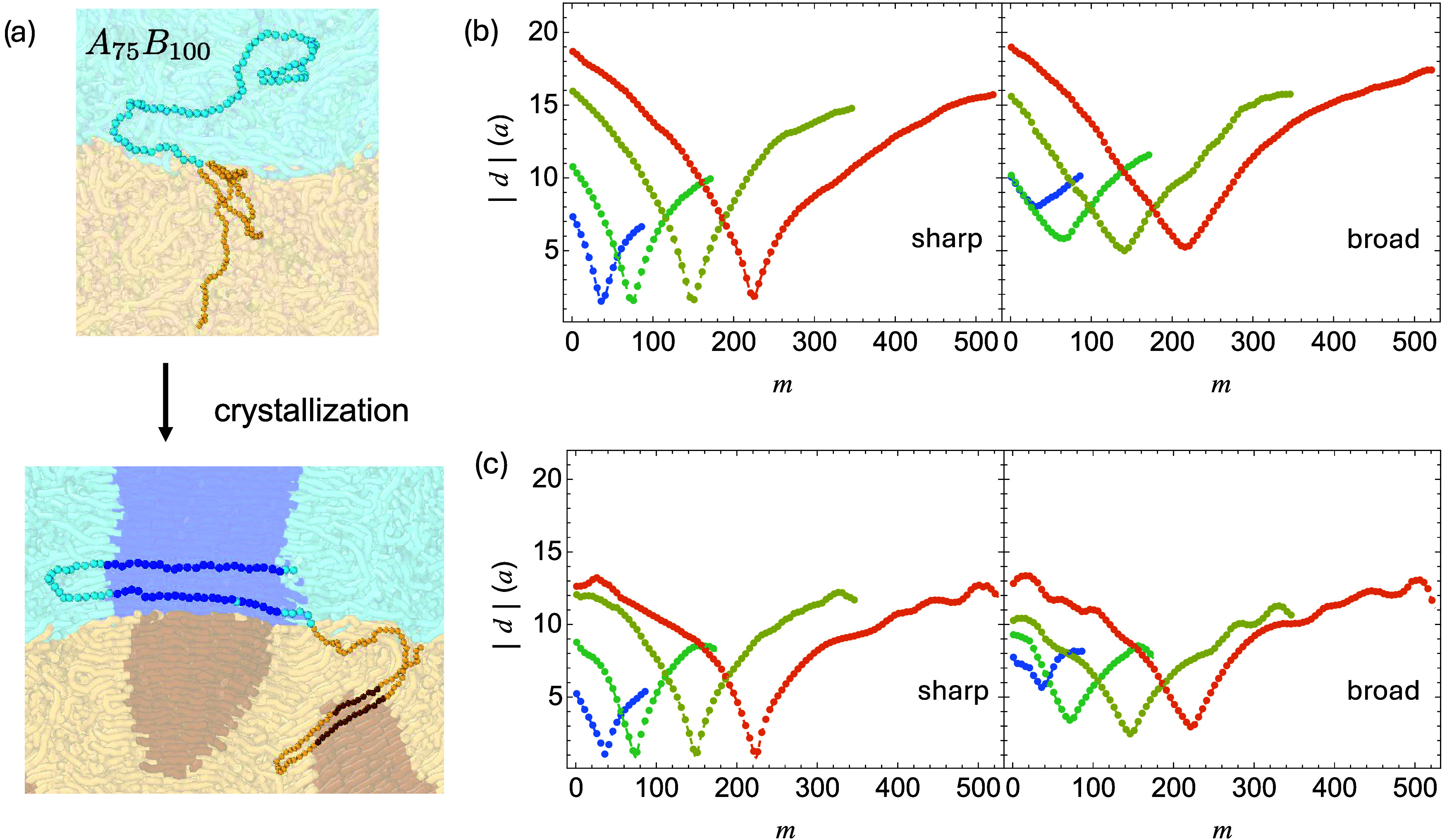
(a) Snapshots of block copolymers at interface before (b) and after crystallization (c). Average absolute distance to the interface |*d*|vs monomer index *m* for block copolymers of different lengths before (b) and after crystallization (c).

To further understand the influence of block copolymers on interfacial morphology prior to crystallization, we analyze the chain conformation and orientation in the molten samples. As shown in Figures S3 and S4, polymer conformations near the interface are anisotropic, indicated by the diagonal components of the gyration tensor. A negative orientational order (*P*_2_(*â* ·*ẑ*)) also reveals chain flattening and preferential alignment parallel to the interface. The interface-induced polymer alignment and packing are less pronounced in the broader interfaces of weakly incompatible blends, consistent with the prediction of self-consistent field theory.^49^ Notably, even under the highest loading of long block copolymers, the addition of compatibilizers does not significantly alter polymer morphology or density profile (Figure S5) near the interface, suggesting that the compatibilizers largely conform to the existing interfacial structure.

Among all tested block copolymers, the shortest *A*_38_ *B*_50_ shows notably different behaviors, especially near broad interfaces. Its junction point tends to reside at the edge of the interface. Its low molecular weight results in a greater translational entropy penalty for localizing the block copolymer in the interfacial region. Furthermore, the block length of *A*_38_ *B*_50_ is shorter than twice the average crystalline stem length, which limits its ability to form folded crystalline stems. Its crystallization pathway may therefore differ from that of longer counterparts and is beyond the scope of the present discussion. We decided to focus on studying the crystallization mechanism of the three longer block copolymers, while results for *A*_38_ *B*_50_ are provided in Supporting Information.

Upon crystallization, long copolymer blocks can be incorporated into crystalline domains formed by homopolymers (Figure 8a), while their junctions are still confined at the interfaces (Figure 8c). Limited polymer relaxation near the center of the block copolymers hinders the overall crystallization in the interfacial region. Across all systems, the introduction of block copolymers reduces crystallinity near the interface, regardless of block copolymer length or loading (Figure 9). As indicated by the distributions of crystallinity near interfaces.

**Figure 9:**
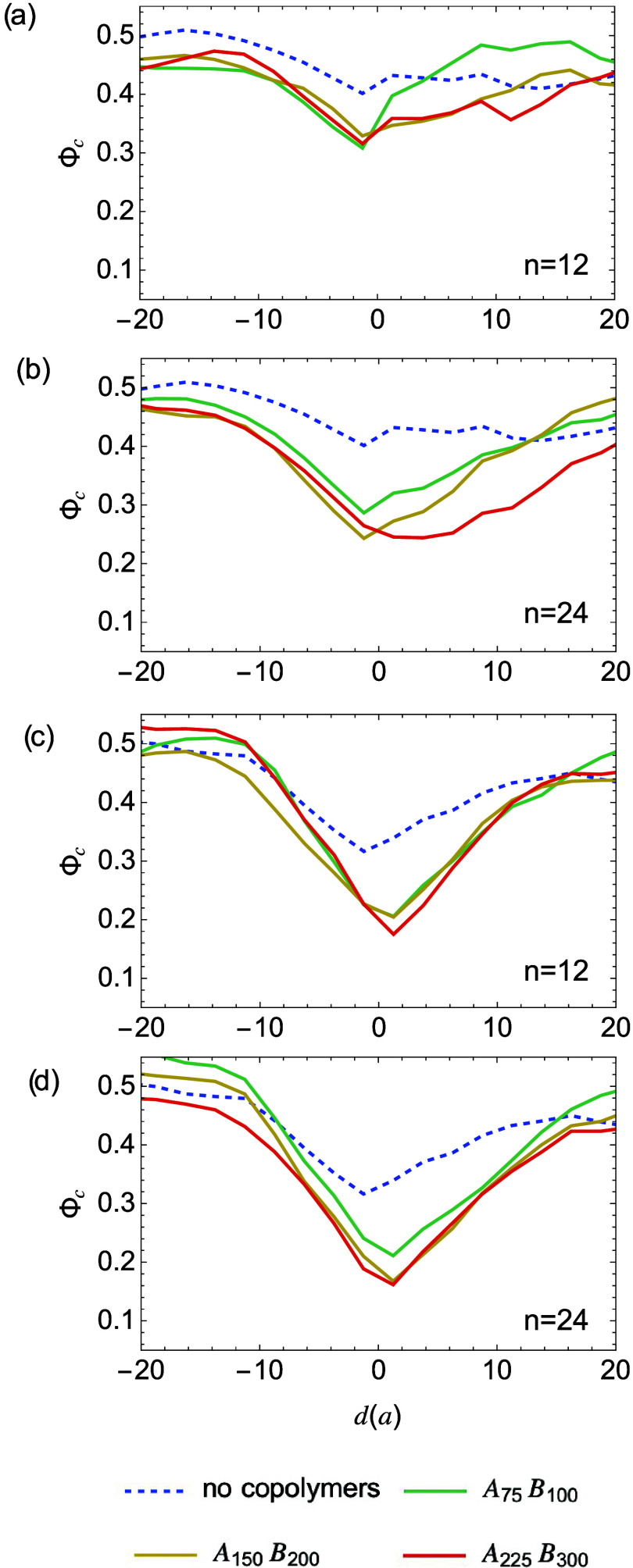
Local crystallinity Φ_c_ vs distance *d* to the sharp interface (a) and (b), and to broad interfaces (c) and (d) under different block copolymer loadings.

The observed reduction in crystallinity near compatibilized interfaces cannot be attributed to morphological change in the molten state, as block copolymer doping does not significantly alter the interfacial packing or density profiles. As demonstrated in Figures S3–S5, the addition of long block copolymers at high concentrations imposes negligible effects on polymer morphologies near the interface. Compositional heterogeneity and packing frustration are unlikely to be the primary cause of suppressed crystallization.

Instead, the reduction in crystallinity may arise from hindered disentanglement dynamics imposed by “tethering” block copolymers to the interface. As previously reported, crystallization is accompanied by disentanglement,^32^ since entanglement kinks are disordered coils and thus incompatible with ordered crystalline regions. As a crystal stem grows, it pushes kinks toward the remaining amorphous region and disentangles if allowed. Reptation allows entanglement strands near chain ends to relax quickly, and thus, monomers near chain ends crystallize faster than central segments. This is supported by chain-wise crystallinity profiles of both homopolymers and block copolymers, which show higher ⟨*S*⟩ values at chain ends (Figure 10a).

**Figure 10:**
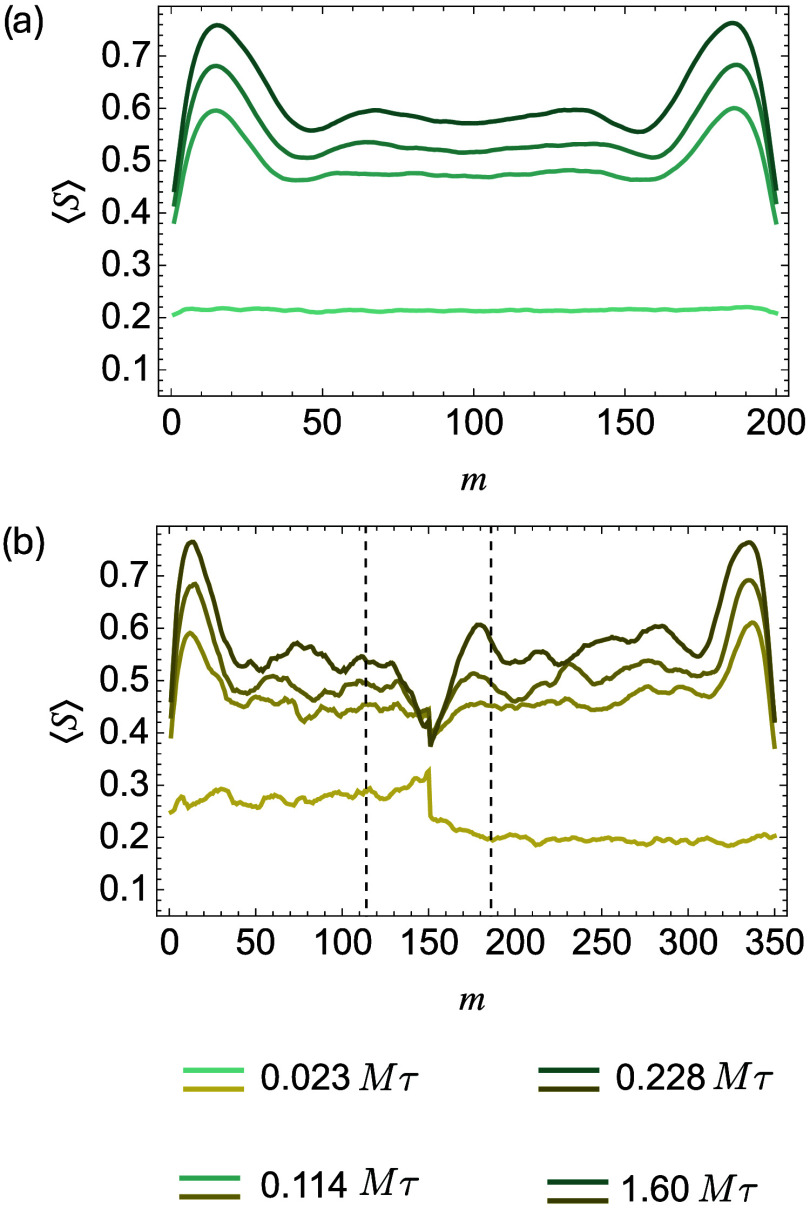
Average order parameter ⟨*S*⟩ vs monomer index *m* for homopolymers (a) and block copolymers (b) in broad interfaces doped with *A*_150_ *B*_200_ (*n* = 12) at different simulation time. The dashed lines mark ± *N*_e_ away from the junction point.

Unlike homopolymers, whose chain ends and centers disperse throughout the sample, the junctions of block copolymers are confined at the interface. The middle sectors of these additives relax slowly, making the interfacial region more sluggish than the interface without compatibilization. Under strong confinement (high incompatibility), the compatibilizers may even act as “two-armed stars”, relaxing near the interface through slow arm retraction and constraint release.^50^ The confinement of block junctions significantly restricts the ability of block copolymers to disentangle with themselves and homopolymers and thus suppresses the growth of crystalline order in the interfacial region, indicated by a pronounced local minimum in ⟨*S*⟩ at the junction point (Figure 10b). For the long block copolymer chains (*A*_150_ *B*_200_ and *A*_225_ *B*_300_), their segments with reduced crystallinity extend over a length approximately equal to the entanglement length *N*_*e*_, calculated using Z1+ as *N*_e_^*A*^ = 36.6 and *N*_e_^*B*^ = 36.0, from the block junctions (marked by dashed lines in Figures 10b and S11–S13). Entanglement strands adjacent to the confined block junctions cannot easily relax and crystallize.

To directly quantify disentanglement near interfaces during crystallization, we use the Z1+ algorithm to track the evolution of entanglement topology during crystallization. Consistent with prior studies,^32^ the total entanglement count exhibits a short-lived increase during initial cooling due to increased chain stiffness and density, followed by a steady decline as crystallization proceeds. We define the entanglement loss as *e*_loss_(*t*) = (*Z*_max_ – Z­(*t*))/*Z*_max_, where *Z*_max_ is the maximum number of entanglement kinks after cooling but before crystallization begins (Figures 11a and S6).

In interfacial regions (|*d*|< 7.5 *a*, where the crystallinity is significantly reduced), samples with block copolymers exhibit lower *e*_loss_ compared to the disentanglement in the same region of uncompatibilized homopolymer blends. To understand the species-specific contributions, we further analyze the disentanglement of homopolymers and block copolymers near interfaces separately (Figures 11b and S7–S8). The loss of entanglement kinks formed by block copolymers is significantly reduced, while the homopolymers retain a similar disentanglement rate compared to those in the uncompatibilized blends. And as expected, increasing the molecular weight of block copolymers impedes entanglement relaxation near the interface.

The presence of entangled block copolymers near the interface also suppresses the crystallization of neighboring homopolymers. Compared to blends without block copolymers, homopolymers in block copolymer-doped systems exhibit significantly lower crystallinity near the interface (Figures 12a and S14). The hindered stem growth of block copolymers also limits the growth of nearby homopolymer crystal stems. As a result, their average crystal stem lengths are shorter compared to those in uncompatibilized samples, evidenced by the stem length distribution near the interface (Figures 12b and S15). Overall, our results suggest that the hindered disentanglement of block copolymers plays a critical role in limiting interfacial crystallization in the compatibilized semicrystalline blends (Figure 12).

**Figure 11:**
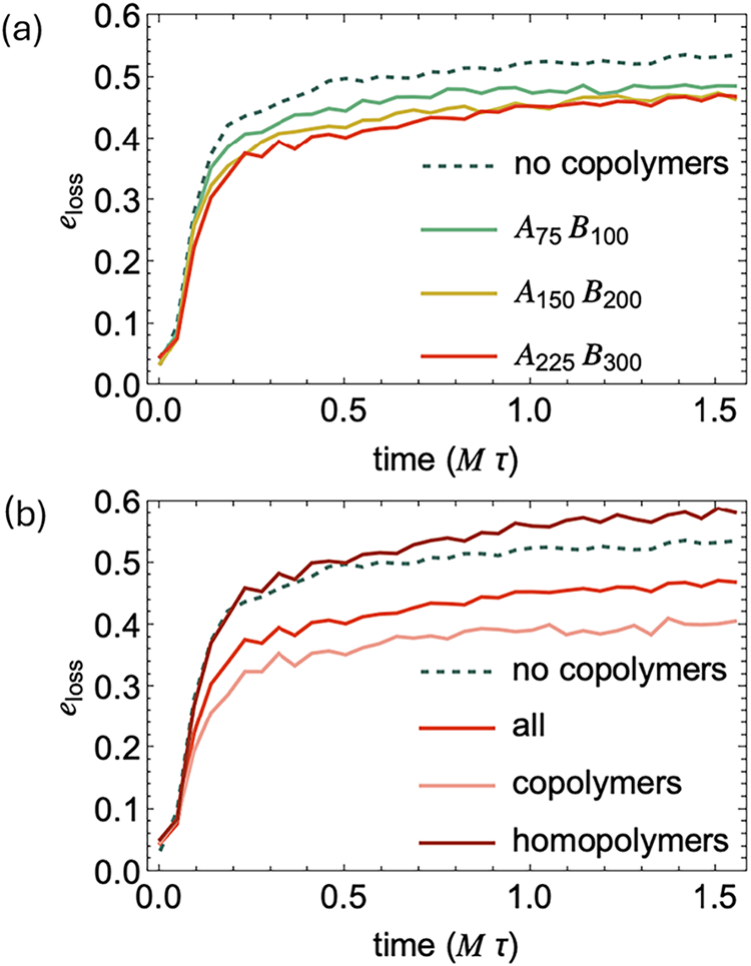
(a) Degree of disentanglement *e*_loss_ vs time for all polymers in the interfacial region (|*d*|< 7.5 *a*) of uncompatibilized blends (dashed curve) and samples with 12 block copolymers per interface (solid curves) during crystallization. (b) Degree of disentanglement vs time for all polymers, block copolymers, and homopolymers in the interfacial region (|*d*|< 7.5 *a*) with 12 *A*_225_ *B*_300_ block copolymers per interface.

**Figure 12:**
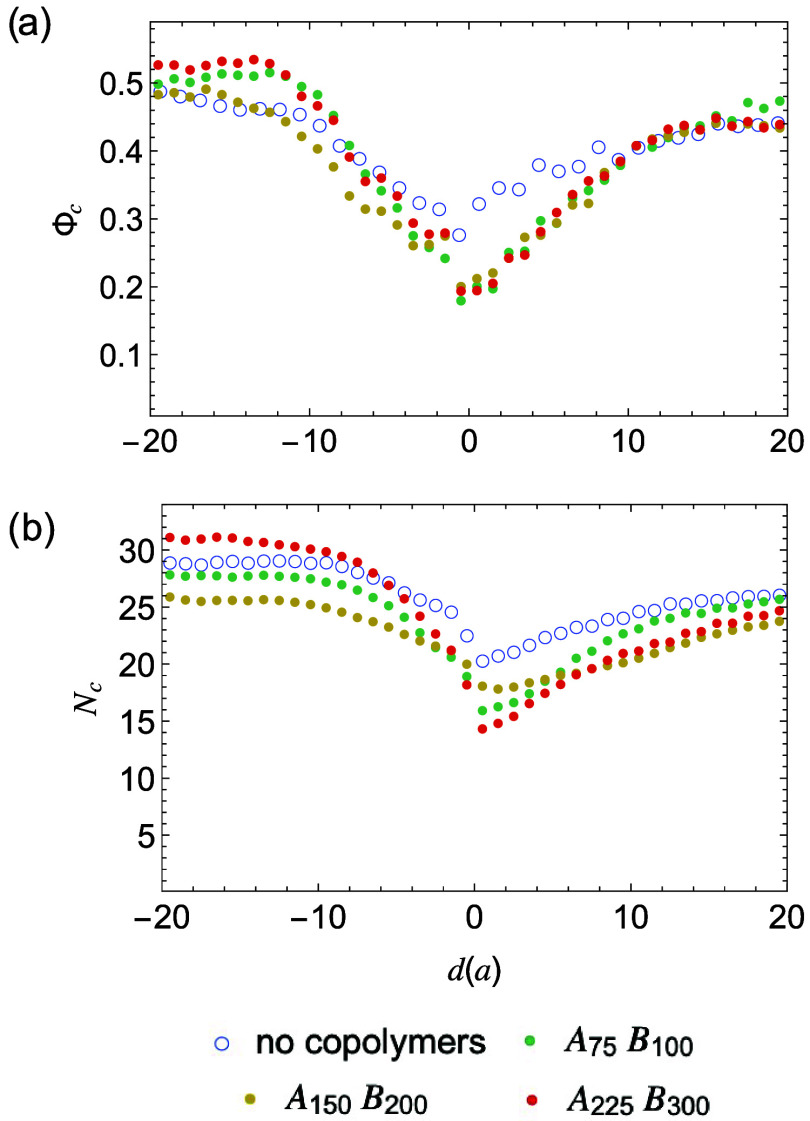
(a) Distributions of local crystallinity Φ_c_ (a) and crystalline stem length *N*_c_ (b) of homopolymers in blends without and with block copolymers (*n* = 12).

The effects of block copolymers on crystallization are qualitatively similar in blends with either sharp or broad interfaces (see Figures 9 and S6–S15). However, the reduction of crystallization is more pronounced in samples with broad interfaces. Homopolymers near sharp interfaces can crystallize more readily due to the lower spatial extent of compositional inhomogeneity. The suppression of crystallization mostly arises from block copolymer-induced slow disentanglement kinetics. Near broad interfaces, homopolymers experience restrictions from both the inhomogeneous interface and the block copolymer-induced slow polymer relaxation, leading to a more pronounced decrease in crystallinity.

Although direct cocrystallization between the two species is prohibited due to their mismatched monomer sizes, block copolymers can crystallize on both sides of the interface and act as tie bridges connecting the two phases. These structures are expected to play a key role in mechanical reinforcement by transmitting stress across the interface when the system is deformed (Figure 13). We find that the fraction of block copolymers that crystallize with both species and form tie bridges increases with block copolymer length, approaching unity when the compatibilizers are sufficiently long—likely when each block exceeds the sum of entanglement length and average crystal stem length *N*_e_ + *N̅*_c_, as crystallization within approximately one *N*_e_ of the junction is significantly restricted, allowing both sides to fully crystallize and bridge the interface. In contrast, the incompatibility and interfacial width of the molten samples have only minor effects on the formation of such bridges (Figure 14).

**Figure 13:**
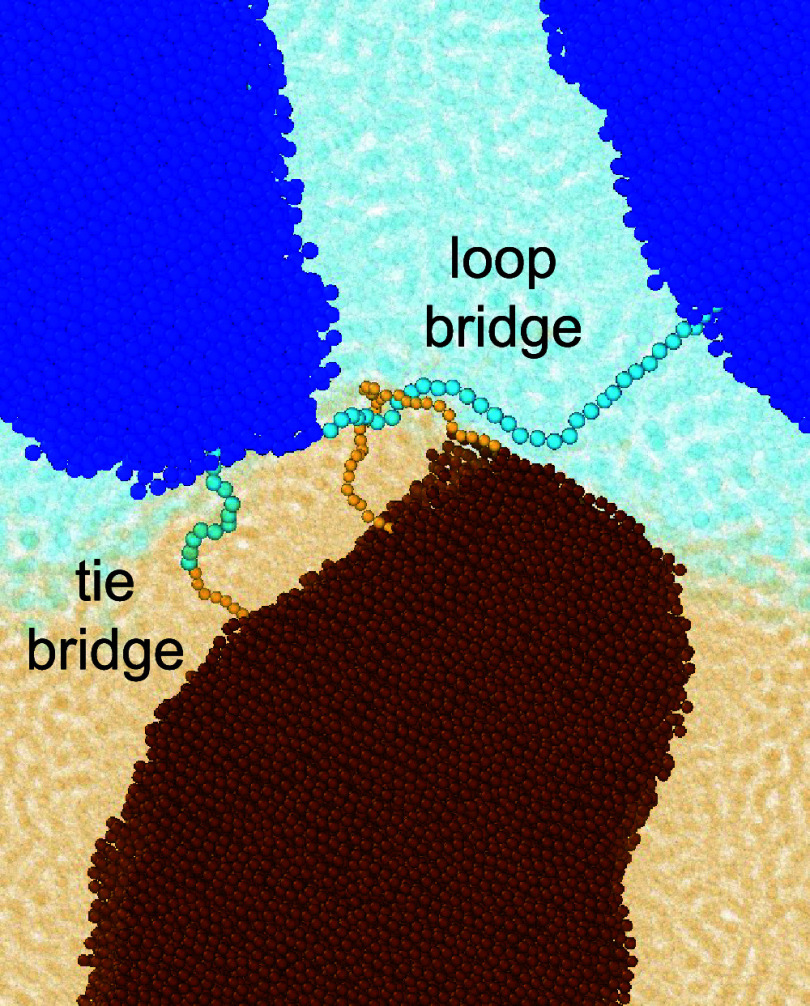
Snapshots of one loop bridge and one tie bridge formed after crystallization of broad interfaces doped with *A*_75_ *B*_100_.

**Figure 14:**
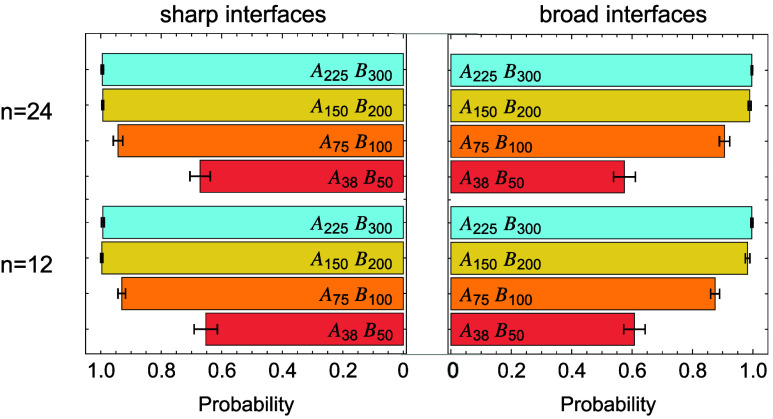
Probability of tie bridge formation by block copolymers of different lengths for different interface types (sharp vs broad) and block copolymer loadings.

Another potential stress transmitter is the entangled loop bridge. This stress transmitter involves two chains originating from opposite sides of the interface that are entangled with one another and have both ends incorporated into crystalline domains. Figure 15 shows the average number of loop bridges formed between the two polymer domains. In sharp interfaces, loop bridges are rare, regardless of whether block copolymers are added, due to the low density of interspecies entanglement near the interface. In contrast, broad interfaces exhibit a higher number of loop bridges due to enhanced mixing in the interfacial region.

**Figure 15:**
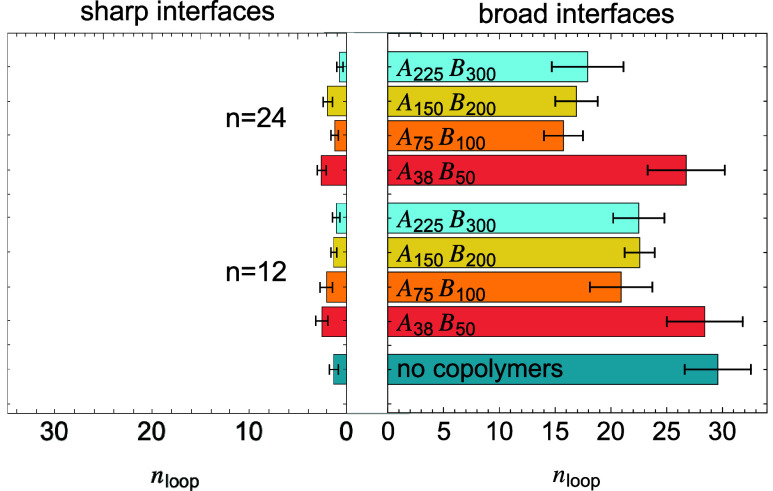
Number of loop bridges near sharp and broad interfaces, with and without block copolymers at different loadings.

Increasing the number of block copolymers at the interface leads to reduced loop bridge formation. This is a result of reduced crystallinity in the interfacial region, as block copolymers “push” crystals away from the interface, thereby lowering the probability of forming entangled loops with both ends crystallized. The mechanical behaviors of tie bridges and loop bridges and how to balance the two to create an optimized interface—remain an open question and will be the subject of future studies.

### Conclusion

In this study, we employ coarse-grained simulations to investigate the crystallization of homopolymers and block copolymer compatibilizers near immiscible polymer interfaces. By adjusting the interspecies interactions, we control incompatibility in polymer blends and create interfaces with different widths. We also introduce mismatches in monomer sizes to avoid artificial cocrystallization in the interfacial region, a process not observed in many realistic semicrystalline systems such as recycled polyolefins.

Our results show that broad interfaces suppress crystallization and limit crystal stem growth across a region significantly wider than the compositional interface. This suppression is not due to entanglement constraints, which are actually reduced near the interface, but rather results from two coupled effects: (1) incompatible chains act as impurities that interfere with the crystallization, and (2) crystallization prohibits rough crystalline surfaces of stems of uneven lengths, limiting the ability of stems to grow near disordered interfacial regions.

The addition of block copolymers further hinders crystallization. The block junctions, confined near the interface, impede nearby disentanglement and crystal growth. By coupling to the compositional inhomogeneity, the hindered entanglement relaxation induced by block copolymers can noticeably suppress crystallization near interfaces in weakly phase-separated blends.

Despite hindering crystallization near blend interfaces, long block copolymers can crystallize within both polymer domains and form tie bridges across the interface, which may provide mechanical reinforcement. To promote the tie bridge formation, we show that the length of the blocks should be greater than the total length of an entanglement strand and a crystalline stem. However, the number of entangled loop bridges can decrease due to reduced crystallization induced by block copolymers near the interface. Balancing the tie and loop bridges could be key to optimizing the performance of block copolymer compatibilizers for semicrystalline blends.

## Supplementary Material



## Notes

Detailed setup of polymer blends and summarized crystallization behaviors of samples doped with different block copolymers (PDF)

## References

[1] Jones, R. A. ; Richards, R. W. Polymers at Surfaces and Interfaces; Cambridge University Press: New York, 1999; pp 127–128.

[2] García-Collado A., Blanco J., Gupta M. K., Dorado-Vicente R. (2022). Advances in
polymers based multi-material additive-manufacturing techniques: state-of-art
review on properties and applications. Addit.
Manuf..

[3] Schyns Z. O. G., Shaver M. P. (2021). Mechanical recycling
of packaging plastics: a review. Macromol. Rapid
Commun..

[4] Yin S., Tuladhar R., Shi F., Shanks R. A., Combe M., Collister T. (2015). Mechanical
reprocessing of polyolefin waste: a review. Polym. Eng. Sci..

[5] Michell R. M., Müller A. J. (2016). Confined crystallization of polymeric materials. Prog. Polym. Sci..

[6] Jordan A. M., Kim K., Soetrisno D., Hannah J., Bates F. S., Jaffer S. A., Lhost O., Macosko C. W. (2018). Role of crystallization on polyolefin
interfaces: an improved outlook for polyolefin blends. Macromolecules.

[7] Matsuba G., Shimizu K., Wang H., Wang Z., Han C. C. (2004). The effect
of phase separation on crystal nucleation density and lamella growth
in near-critical polyolefin blends. Polymer.

[8] Arai F., Takeshita H., Dobashi M., Takenaka K., Miya M., Shiomi T. (2012). Effects of
liquid-liquid phase separation on crystallization
of poly­(ethylene glycol) in blends with isotactic poly (methyl methacrylate). Polymer.

[9] Jin J., Chen H., Muthukumar M., Han C. C. (2013). Kinetics pathway
in the phase separation and crystallization of *i*PP/OBC
blends. Polymer.

[10] Ma Y., Zha L., Hu W., Reiter G., Han C. C. (2008). Crystal nucleation
enhanced at the diffuse interface of immiscible polymer blends. Phys. Rev. E:Stat., Nonlinear, Soft Matter Phys..

[11] Mitra M. K., Muthukumar M. (2010). Theory of spinodal decomposition
assisted crystallization
in binary mixtures. J. Chem. Phys..

[12] Zhang W., Zou L. (2021). Molecular dynamics simulations of crystal nucleation near interfaces
in incompatible polymer blends. Polymers.

[13] Huang D. E., Kotula A. P., Snyder C. R., Migler K. B. (2022). Crystallization
kinetics in an immiscible polyolefin Blend. Macromolecules.

[14] Zhang W., Zou L. (2023). Mismatch in nematic interactions
leads to composition-dependent crystal
nucleation in polymer blends. Macromolecules.

[15] Jose S., Aprem A. S., Francis B., Chandy M. C., Werner P., Alstaedt V., Thomas S. (2004). Phase morphology,
crystallisation
behaviour and mechanical properties of isotactic polypropylene/high
density polyethylene blends. Eur. Polym. J..

[16] Derakhshandeh M., Doufas A. K., Hatzikiriakos S. G. (2014). Quiescent
and shear-induced crystallization
of polyprophylenes. Rheol. Acta.

[17] Mi D., Xia C., Jin M., Wang F., Shen K., Zhang J. (2016). Quantification
of the effect of shish-kebab structure on the mechanical properties
of polypropylene samples by controlling shear layer thickness. Macromolecules.

[18] Coughlin M. L., Huang D. E., Edgar C. M., Kotula A. P., Migler K. B. (2024). Composition
dependence of flow-induced crystallization in high-density polyethylene/isotactic
polypropylene blends. Macromolecules.

[19] Ma Y., Hu W., Reiter G. (2006). Lamellar crystal
orientations biased by crystallization
kinetics in polymer thin films. Macromolecules.

[20] Luo C., Kröger M., Sommer J.-U. (2017). Molecular dynamics simulations of
polymer crystallization under confinement: Entanglement effect. Polymer.

[21] Ramos P. M., Herranz M., Martinez-Fernandez D., Foteinopoulou K., Laso M., Karayiannis N. C. (2022). Crystallization
of flexible chains
of tangent hard spheres under full confinement. J. Phys. Chem. B.

[22] Ming Y., Hao T., Zhou Z., Zhang S., Nie Y. (2023). Study on the crystallization
behavior of polymer thin films with a substrate by computer simulation. Cryst. Growth Des..

[23] Benkoski J. J., Flores P., Kramer E. (2003). Diblock copolymer reinforced interfaces
between amorphous polystyrene and semicrystalline polyethylene. Macromolecules.

[24] Tang X., Liu C., Keum J., Chen J., Dial B. E., Wang Y., Tsai W.-Y., Bras W., Saito T., Bowland C. C., Chen X. C. (2022). Upcycling of semicrystalline polymers by compatibilization:
mechanism and location of compatibilizers. RSC
Adv..

[25] Eagan J. M., Xu J., Di Girolamo R., Thurber C. M., Macosko C. W., LaPointe A. M., Bates F. S., Coates G. W. (2017). Combining polyethylene and polypropylene:
enhanced performance with PE/*i*PP multiblock polymers. Science.

[26] Tang X., Liu C., Chen J., Kumar R., Bowland C. C., Saito T., Dial B. E., Keum J. K., Do C., Chen X. C. (2024). Probing
the interface structure of block copolymer compatibilizers in semicrystalline
polymer blends. J. Appl. Polym. Sci..

[27] Xu J., Eagan J. M., Kim S.-S., Pan S., Lee B., Klimovica K., Jin K., Lin T.-W., Howard M. J., Ellison C. J. (2018). Compatibilization of isotactic polypropylene
(*i*PP) and high-density polyethylene (HDPE) with *i*PP–PE multiblock copolymers. Macromolecules.

[28] López-Barrón C. R., Tsou A. H. (2017). Strain hardening
of polyethylene/polypropylene blends
via interfacial reinforcement with poly­(ethylene-cb-propylene) comb
block copolymers. Macromolecules.

[29] Moghanlou S., Khamseh M., Razavi Aghjeh M., Pourabbas B. (2020). Influence
of chain extension and blending on crystallinity and morphological
behavior of recycled-PET/ethylene vinyl acetate blends. J. Polym. Environ..

[30] Ferri J. M., Garcia-Garcia D., Rayón E., Samper M. D., Balart R. (2020). Compatibilization
and characterization of polylactide and biopolyethylene binary blends
by non-reactive and reactive compatibilization approaches. Polymers.

[31] Hess B., Kutzner C., Van Der Spoel D., Lindahl E. (2008). GROMACS 4: algorithms
for highly efficient, load-balanced, and scalable molecular simulation. J. Chem. Theory Comput..

[32] Zhai Z., Fusco C., Morthomas J., Perez M., Lame O. (2019). Disentangling
and lamellar thickening of linear polymers during crystallization:
simulation of bimodal and unimodal molecular weight distribution systems. ACS Nano.

[33] Zhai Z., Morthomas J., Fusco C., Perez M., Lame O. (2019). Crystallization
and molecular topology of linear semicrystalline polymers: simulation
of uni-and bimodal molecular weight distribution systems. Macromolecules.

[34] Mencik Z. (1972). Crystal structure
of isotactic polypropylene. J. Macromol. Sci.,
Part B.

[35] Alsaygh A. A., Al-hamidi J., Alsewailem F. D., Al-Najjar I. M., Kuznetsov V. L. (2014). Characterization of polyethylene synthesized by zirconium
single site catalysts. Appl. Petrochem. Res..

[36] Morthomas J., Fusco C., Zhai Z., Lame O., Perez M. (2017). Crystallization
of finite-extensible nonlinear elastic Lennard-Jones coarse-grained
polymers. Phys. Rev. E.

[37] Hoy R. S., Karayiannis N. C. (2013). Simple
model for chain packing and crystallization
of soft colloidal polymers. Phys. Rev. E.

[38] Nguyen H. T., Smith T. B., Hoy R. S., Karayiannis N. C. (2015). Effect
of chain stiffness on the competition between crystallization and
glass-formation in model unentangled polymers. J. Chem. Phys..

[39] Helfand E., Sapse A. M. (1975). Theory of unsymmetric
polymer-polymer interfaces. J. Chem. Phys..

[40] Strobl G. (2009). Colloquium:
Laws controlling crystallization and melting in bulk polymers. Rev. Mod. Phys..

[41] Parrinello M., Rahman A. (1981). Polymorphic transitions in single
crystals: a new molecular
dynamics method. J. Appl. Phys..

[42] Kröger M., Dietz J. D., Hoy R. S., Luap C. (2023). The Z1+ package: shortest
multiple disconnected path for the analysis of entanglements in macromolecular
systems. Comput. Phys. Commun..

[43] Jeon H. S., Lee J., Balsara N. (1998). Predictions
of the thermodynamic properties of multicomponent
polyolefin blends from measurements on two-component systems. Macromolecules.

[44] Hiemenz, P. C. ; Lodge, T. P. Polymer Chemistry; CRC Press, 2007; p 224.

[45] Ge T., Grest G. S., Robbins M. O. (2013). Structure
and strength at immiscible
polymer interfaces. ACS Macro Lett..

[46] Zhang Y., Zhang W. (2025). Effects of block copolymer
compatibilizers and interfacial entanglements
on strengthening immiscible glassy polymer blends. Macromolecules.

[47] Zou L., Zhang W. (2024). Effects of entanglement on polymer crystal growth and
intercrystalline
phase formation. Macromolecules.

[48] Peng F., Cao R., Sun H., Liu Z., Zhang Y., Xu T., Li L. (2024). Role of entanglement
in polymer crystal growth and melting: molecular
dynamics simulations. Macromolecules.

[49] Morse D. C., Fredrickson G. H. (1994). Semiflexible
polymers near interfaces. Phys. Rev. Lett..

[50] Larson, R. G. The Structure and Rheology of Complex Fluids; Oxford University Press: New York, 1999; Vol. 150, pp 169–170.

