# Supplementary material for: Coarse-Grained Simulations of Crystallization in Phase-Separated Polymer Blends with Block Copolymer Compatibilizers
Source: Macromolecules. 2025 Nov 21;58(23):12871–81. doi: 10.1021/acs.macromol.5c01767 (PMC12874652; doi:10.1021/acs.macromol.5c01767)
Supplement: Supplementary file 1 [file ma5c01767_si_001.pdf]

Coarse-grained simulations of crystallization in phase-separated polymer blends with block copolymer compatibilizers

Yunjia Zhang and Wenlin Zhang*

Department of Chemistry, Dartmouth College, 41 College St. Hanover, NH, USA

E-mail: Wenlin.Zhang@dartmouth.edu

Supporting Information

I. Setup of semicrystalline polymer interfaces

To estimate the melting temperature T_m, we perform heating simulations on the bulk crystalline states of polymers A and B, which were previously prepared via quenching at a rate of $3.65 \times 10^{-7}\, u/(k_B \cdot \tau)$ (see *Methods*). Heating is conducted at various rates, and the onset of melting is identified by the abrupt drop in density, as shown in Figure S1. From these curves, the apparent melting temperatures are extracted as $T_m = 3.1\, u/k_B$ for A and $T_m = 2.8\, u/k_B$ for B, respectively.

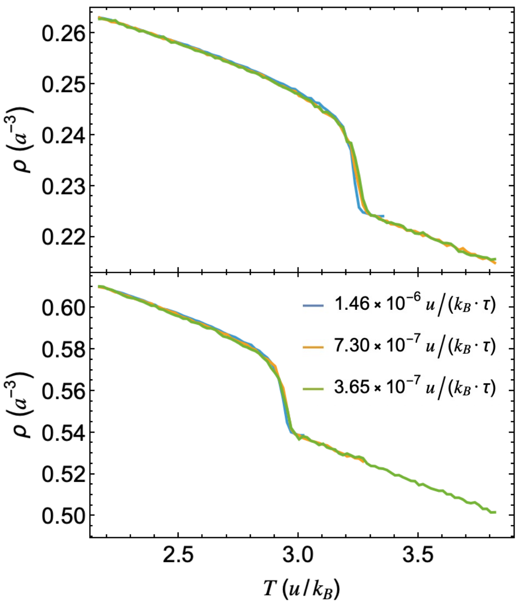

Figure S1: ρ vs. T at different heating rates for polymers A (top) and B (bottom).

To construct homopolymer interfaces, polymers A and B are first equilibrated separately between two flat, impenetrable walls and are then welded together to form a bilayer system. The resulting interfaces are equilibrated to form sharp or broad interfaces. To evaluate the interfacial width and the Flory–Huggins interaction parameter χ, we fit the relative density profile to the asymmetric Helfand–Tagami theory. The density profile is described by:

$$d = \frac{1}{2\alpha^{1/2}} \left[\beta_A \ln \left(\frac{\beta_A - [\beta_A^2(1-\tilde{\rho_A}) + \beta_B^2]^{1/2}}{\beta_A + [\beta_A^2(1-\tilde{\rho_A}) + \beta_B^2]^{1/2}} \times \frac{\beta_A + (1/2\beta_A^2 + 1/2\beta_B^2)^{1/2}}{\beta_A - (1/2\beta_A^2 + 1/2\beta_B^2)^{1/2}} \right) - \frac{1}{2}\beta_B \ln \left(\frac{[\beta_A^2(1-\tilde{\rho_A}) + \beta_B^2]^{1/2} - \beta_B}{[\beta_A^2(1-\tilde{\rho_A}) + \beta_B^2]^{1/2} - \beta_B} \times \frac{(1/2\beta_A^2 + 1/2\beta_B^2)^{1/2} + \beta_B}{(1/2\beta_A^2 + 1/2\beta_B^2)^{1/2} - \beta_B} \right) \right] \quad (1)$$

Here, $\beta_i^2 = \frac{1}{6}\rho_i^0 b_i^2$, where ρ_i^0 is the pure melt density of species i, and b_i is its statistical segment length. $\tilde{\rho}_A = \rho_A/\rho_A^0$ is the volume density of polymer A, the volume density of polymer B is then given by $1 - \tilde{\rho_A}$. From the fitted parameter α, the interfacial width w and Flory–Huggins interaction parameter χ can be calculated as:

$$w = 2\left(\frac{\beta_A^2 + \beta_B^2}{2\alpha}\right)^{1/2} \tag{2}$$

$$\chi = \frac{\alpha}{(\rho_A^0 \rho_B^0)^{1/2}} \tag{3}$$

For the sharp and broad melt interfaces, the fitted interfacial widths are $w = 2.28$a and $w = 9.60$a, respectively. The corresponding Flory–Huggins interaction parameters are $\chi = 0.417$ for the sharp interface and $\chi = 0.027$ for the broad interface. The melt interfaces are then quenched, during which the interfaces narrow and plateau at a narrower interfacial width (Figure S2).

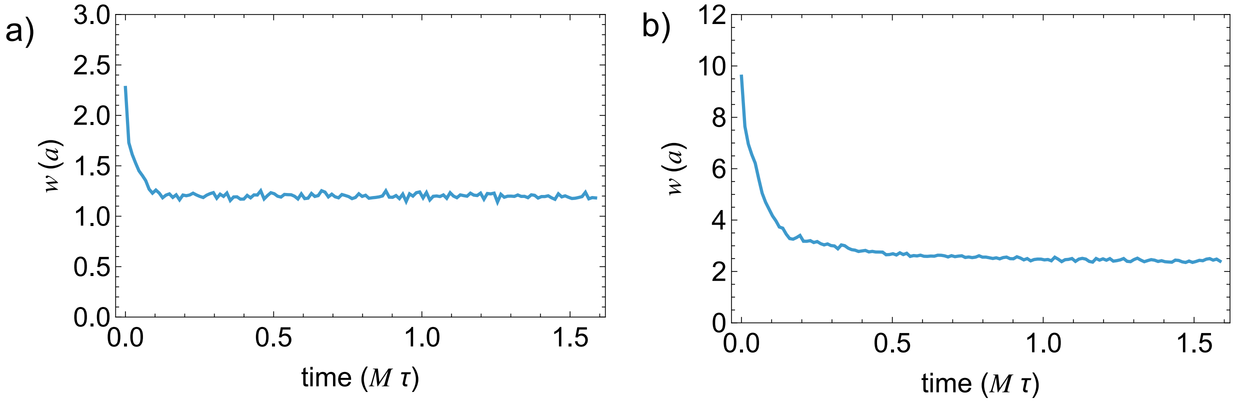

Figure S2: Evolution of calculated interfacial width vs. time for sharp (a) and broad (b) interfaces.

To evaluate influence of spatial correlation on stem length at broad interfaces, we write a phenomenological free energy functional of the normalized stem length distribution $\tilde{N}_c(r) = (N_c(r) - \bar{N}_c)/\bar{N}_c$, where $N_c(r)$ is the local stem length and $\bar{N}_c$ is the bulk average:

$$\frac{F}{k_{\mathrm{B}}T} = \int dr \left[\frac{k_i}{2}\left(\frac{\partial \tilde{N}_c}{\partial r}\right)^2 + f_i(\tilde{N}_c)\right] \tag{4}$$

in which the first term is the surface free energy density that penalizes spatial variation in

stem length. The stiffness constant k_i is of order d_i^2, reflecting the free energy penalty for varying the adjacent stem length by one across one monomer of size d_i is about $k_{\mathrm{B}}T$. The second term, $f_i(\tilde{N}_c)$, describes the bulk free energy density associated with stem growth. We assume that $f_i(\tilde{N}_c)$ is minimized at $\tilde{N}_c = 0$ (bulk equilibrium stem length), rises steeply for $\tilde{N}_c > 0$ due to entanglement constraints, and grows moderately as $\tilde{N}_c$ decreases below zero due to insufficient crystallization and approximate the free energy using a Landau expansion:

$$f_i(\tilde{N}_c) = \alpha_i \tilde{N}_c^{\,2} + \beta_i \tilde{N}_c^{\,3} \tag{5}$$

Here, α_i and β_i represent the bulk free energy densities associated with changing the crystal stem length for species i. The polynomial terms in the Landau expansion asymmetrically penalize deviations from the preferred stem length and we truncate the expansion at the lowest order to permit a simple analytical solution to Euler's equation after functional minimization with respect to $\tilde{N}_c(r)$:

$$\tilde{N}_c(r) = \sqrt{\frac{\alpha_i}{\beta_i}} \left[\tanh^2 \left(\sqrt{\frac{\alpha_i}{2k_i}}\, r \right) - 1 \right] \tag{6}$$

The fitting parameters $\lambda_i = \sqrt{\frac{\alpha_i}{\beta_i}}$ and $\xi_i = \sqrt{\frac{\alpha_i}{2k_i}}$ are defined an used to fit the stem length profile.

Interfaces containing block copolymers are prepared using a similar procedure. Species A and B are first equilibrated separately between confining walls. A thin layer of copolymers is then inserted between the two phases, resulting in a four-layered structure: A–copolymers–B–copolymers. These layers are "welded" together and further equilibrate to produce the final interface. To accommodate the added block copolymers, the number of homopolymer chains in both A and B phases is reduced accordingly. The specific number of each type of polymer used in our simulations is summarized in Table S1.

Table S1: Summarized compositions of block copolymer-doped blends

block copolymer	copolymer number	homopolymer A number	homopolymer B number
/	0	150	300
$A_{38}B_{50}$	24	145	294
$A_{75}B_{100}$	24	141	288
$A_{150}B_{200}$	24	132	276
$A_{225}B_{300}$	24	123	264
$A_{38}B_{50}$	48	141	288
$A_{75}B_{100}$	48	132	276
$A_{150}B_{200}$	48	114	252
$A_{225}B_{300}$	48	96	228

II. Properties of semicrystalline polymer interfaces

In our previous work, we found that adding block copolymers in immiscible melt interfaces results in limited change to the chain packing in the interfacial regions. To evaluate how block copolymers affect interfacial structure, we analyzed chain conformation and orientation in the melt using the diagonal components of the gyration tensor and the orientational order parameter $P_2(\hat{a} \cdot \hat{z})$, where $\hat{a}$ is the the unit backbone tangent vector across a monomer and $\hat{z}$ is the interface normal.

As shown in Figures S3 and S4, chains near the interface exhibit reduced Λ_{zz} and strongly negative P_2, indicating flattened conformations and preferential alignment parallel to the interface. These effects are more pronounced near sharper interfaces.

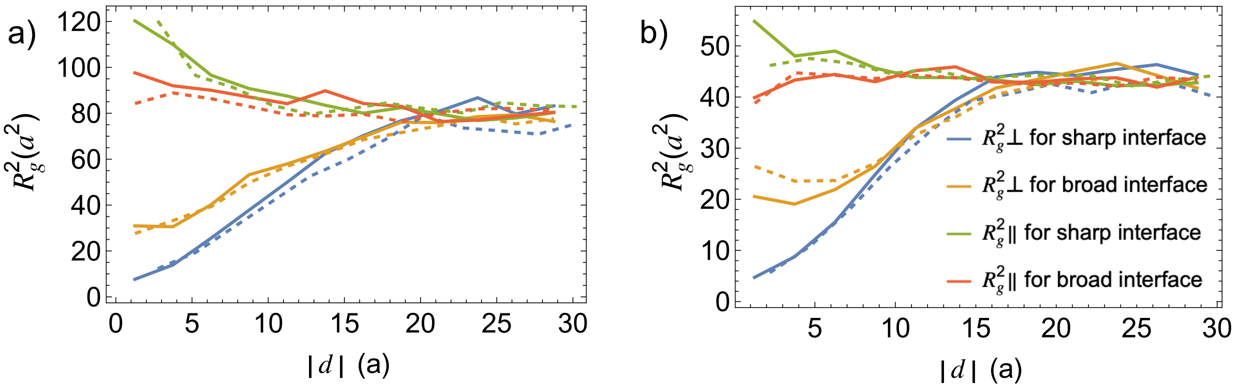

Figure S3: Diagonal components of the gyration tensor of polymer chains in the melt: $R_g^2\perp = \mathbf{\Lambda}_{zz}$ and $R_g^2\| = (\mathbf{\Lambda}_{xx} + \mathbf{\Lambda}_{yy})/2$ vs. distance to the interface for polymer A (a) and polymer B (b), in systems with (dashed lines) and without (solid lines) block copolymer compatibilizers.

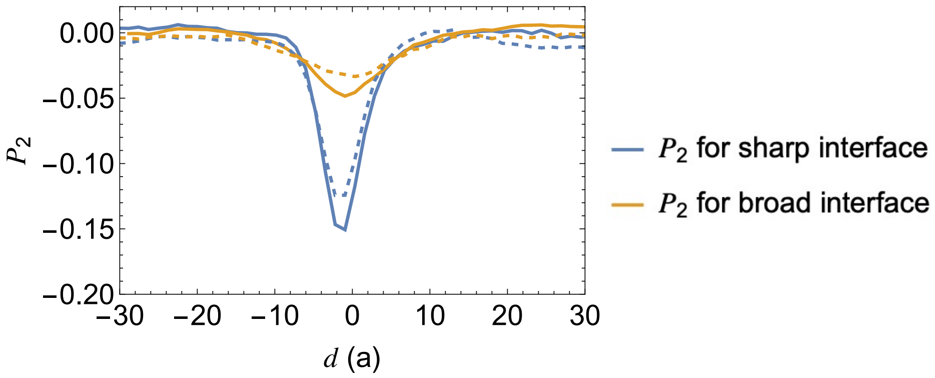

Figure S4: $P_2(\hat{a} \cdot \hat{z})$ of polymer chains in the melt, vs. distance to the interface, for systems with (dashed lines) and without (solid lines) block copolymer compatibilizers.

We examined the system doped with 24 long $A_{225}B_{300}$ block copolymers per interface—the highest loading used—to assess the maximal influence of compatibilizers. The dashed lines show that copolymer addition causes only minor changes in both conformation and orientation. As a result the density profile after adding block copolymers remain mostly the same. This confirms our previous finding that copolymers largely conform to the existing interfacial morphology without significantly disrupting chain packing.

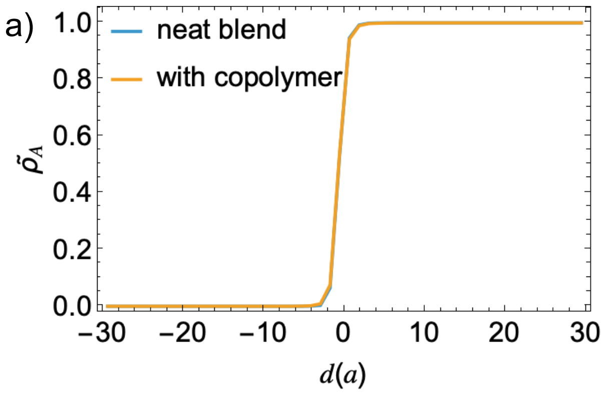

Figure S5: Volume density profile of polymer A ($\tilde{\rho}_A$) in the melt vs. distance to the interface, for (a) sharp and (b) broad interfaces, with and without block copolymer compatibilizers.

Z1+ allows us to track the number of entanglement kinks associated with each individual chain. This enables us to compute the entanglement loss over time, defined as $e_{\text{loss}}(t) = (Z_{\max} - Z(t))/Z_{\max}$, where $Z_{\max}$ is the maximum number of entanglement kinks after cooling but before crystallization begins. In Figure S6, we compare the entanglement loss for all polymers located within the interfacial region ($|d| < 7.5a$). The blue dashed line represents homopolymer systems without copolymers, while solid lines correspond to all in the copolymer-doped systems. Figure S7 and Figure S8 further separate the contributions from homopolymers and block copolymers.

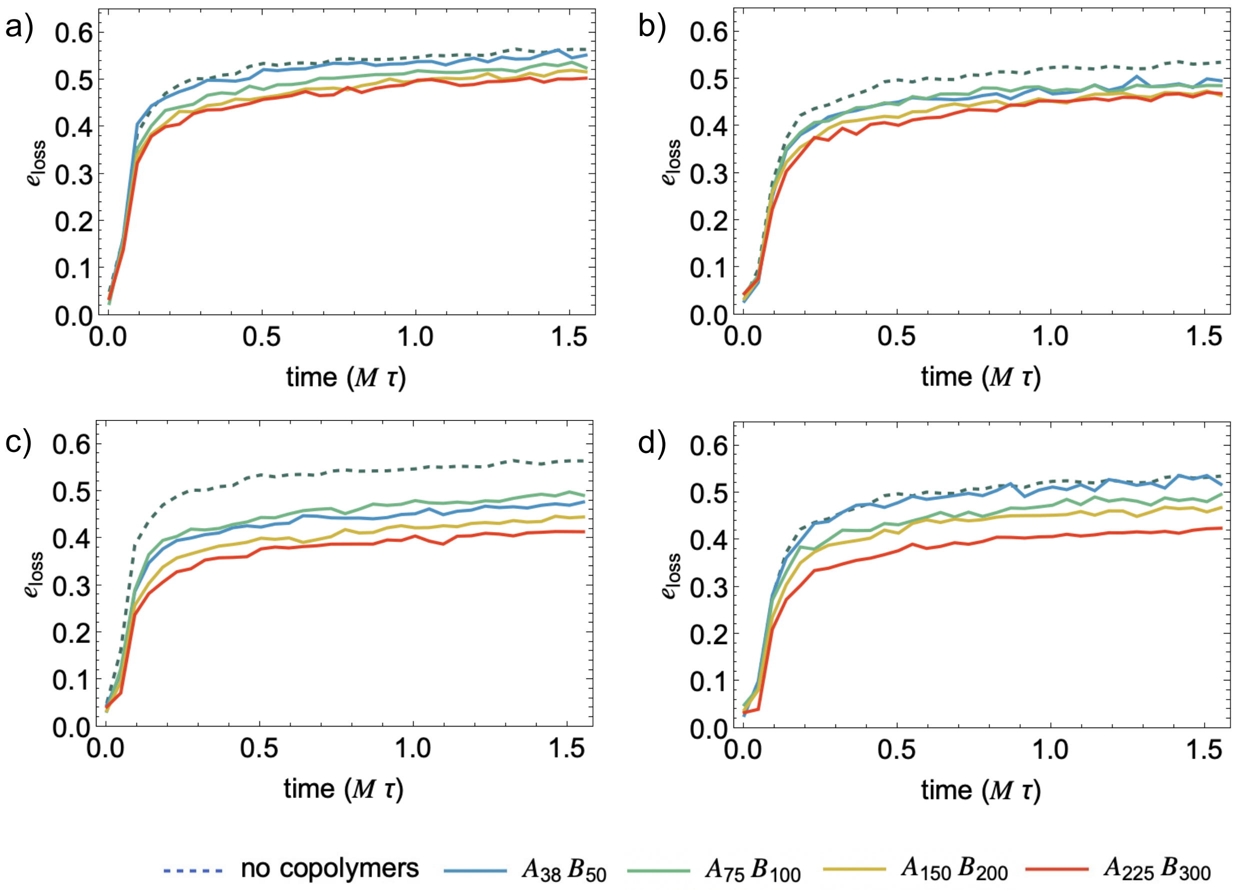

Figure S6: Degree of disentanglement, e_{loss}, vs. time for all polymers in the interfacial region ($|d| < 7.5a$) during crystallization. Results are shown for systems without copolymers (blue dashed line) and with different copolymer loadings: $n = 12$ in (a, b) and $n = 24$ in (c, d), at the sharp (a, c) and broad (b, d) interfaces.

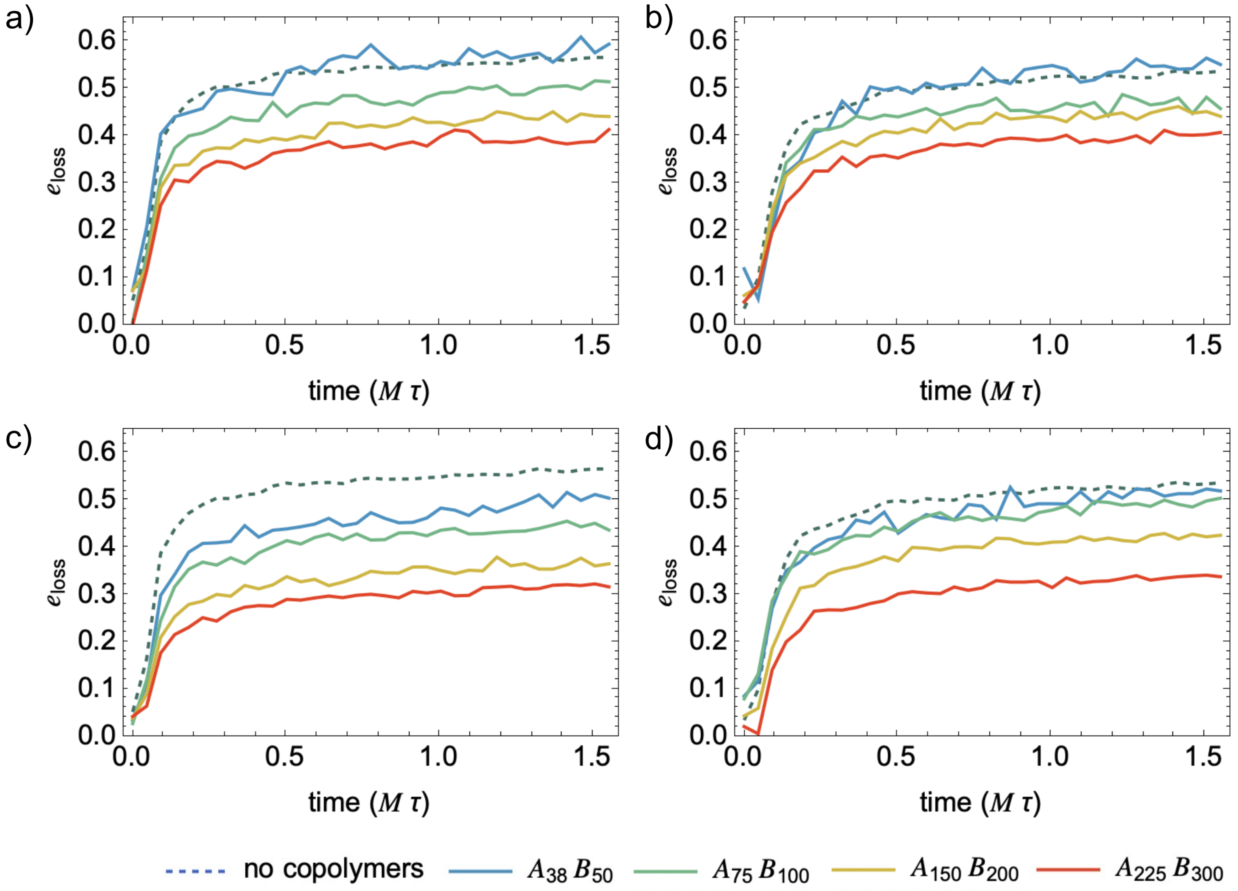

Figure S7: Degree of disentanglement, e_{loss}, vs. time for block copolymers in the interfacial region ($|d| < 7.5a$) during crystallization. Results are shown for systems without copolymers (blue dashed line) and with different copolymer loadings: $n = 12$ in (a, b) and $n = 24$ in (c, d), at the sharp (a, c) and broad (b, d) interfaces.

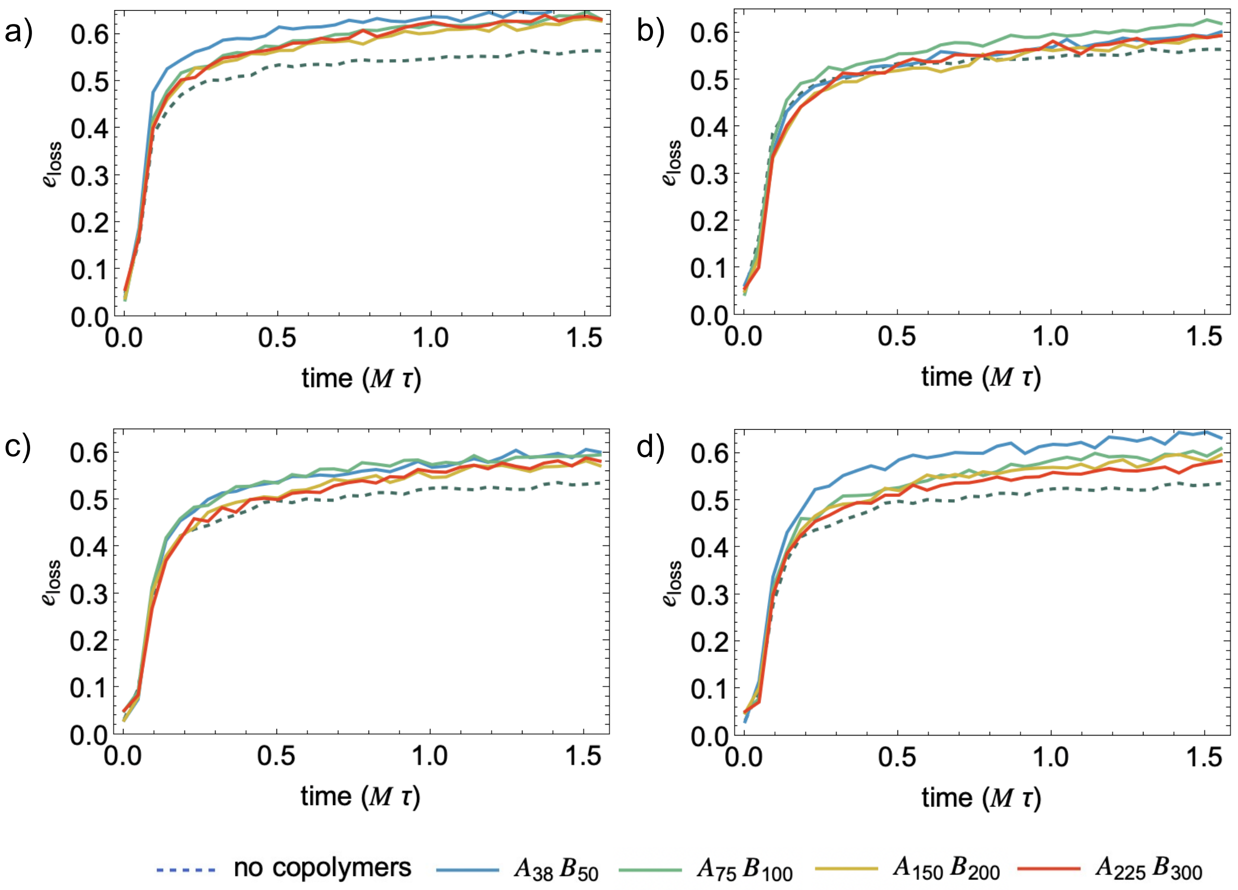

Figure S8: Degree of disentanglement, e_{loss}, vs. time for homopolymers in the interfacial region ($|d| < 7.5a$) during crystallization. Results are shown for systems without copolymers (blue dashed line) and with different copolymer loadings: $n = 12$ in (a, b) and $n = 24$ in (c, d), at the sharp (a, c) and broad (b, d) interfaces.

The orientational order parameter S was calculated from the largest eigenvalue of the local nematic order tensor Q, as described in the main text. Atoms with $S > 0.8$ were classified as crystalline. Based on this criterion, we compute the local crystallinity Φ_c—defined as the fraction of crystalline atoms—vs. distance from the interface (Figure S9). Crystallinity is also evaluated separately for different molecular species such as homopolymers alone (Figure S14). To assess spatial variations in crystallinity along block copolymer chains, we analyze the order parameter S directly for each bead index (Figure S10, S11, S12, and S13). This approach provides better statistical resolution than threshold-based classification, particularly for identifying gradual changes in crystallinity near the copolymer junction.

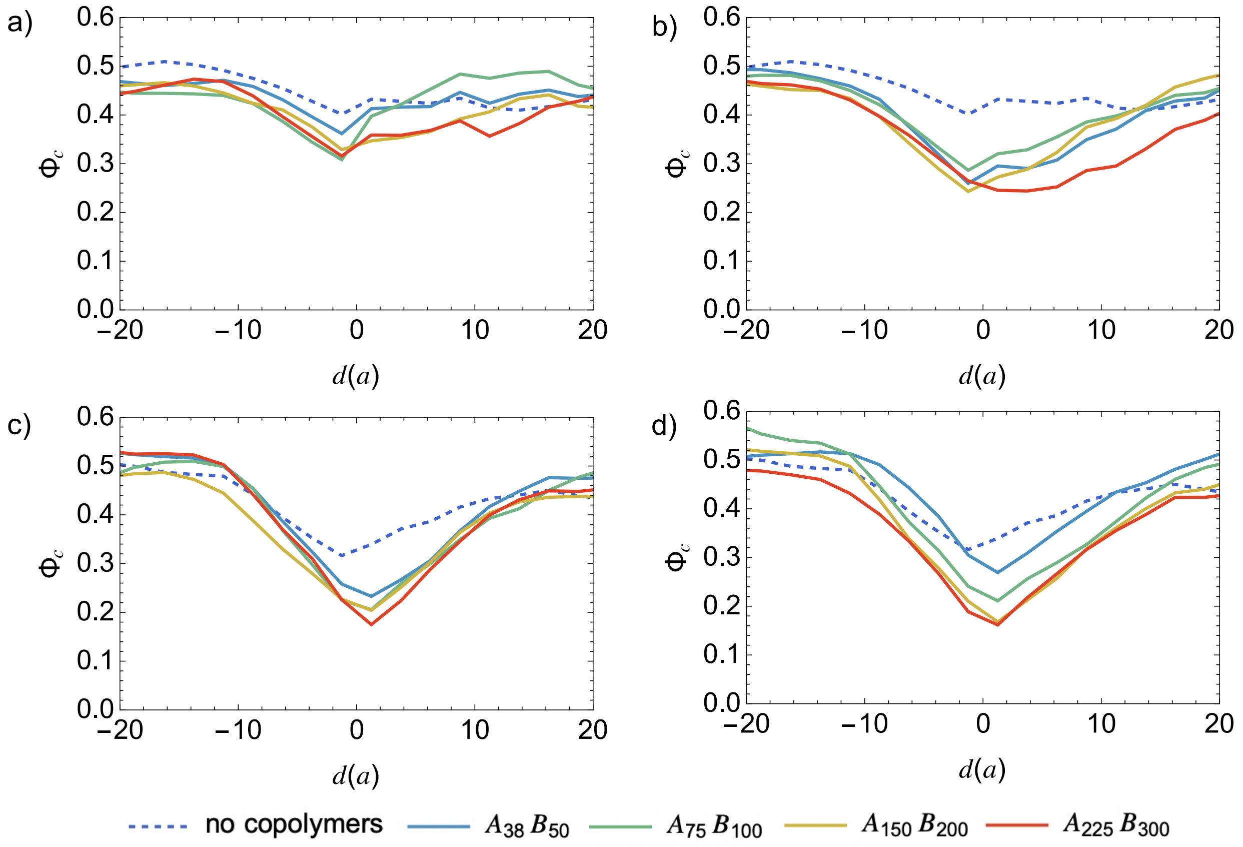

Figure S9: Crystallinity Φ_c vs. distance to the interface for sharp interfaces in (a) and (b), and broad interfaces in (c) and (d), under different copolymer loadings.

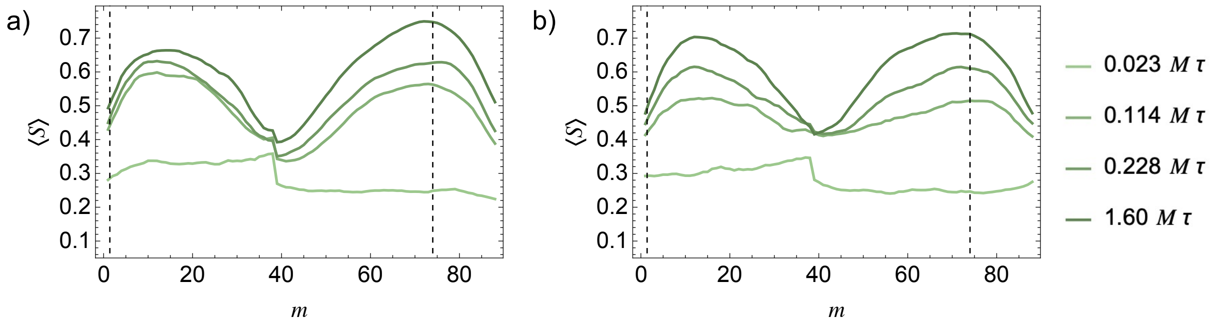

Figure S10: Average order parameter $\langle S \rangle$ vs. monomer index m for block copolymers in broad (a) and sharp (b) interfaces doped with $A_{38}B_{50}$ (n=12). The dashed lines mark $\pm N_e$ away from the junction point.

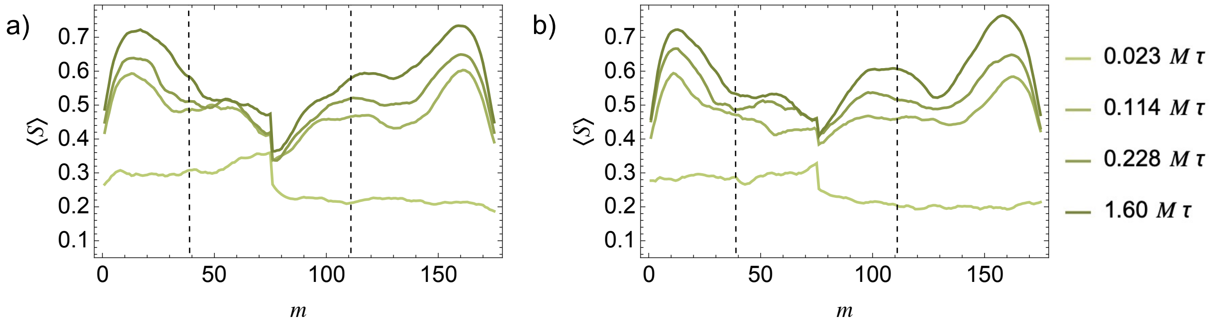

Figure S11: Average order parameter $\langle S \rangle$ vs. monomer index m for block copolymers in broad (a) and sharp (b) interfaces doped with $A_{75}B_{100}$ (n=12). The dashed lines mark $\pm N_e$ away from the junction point.

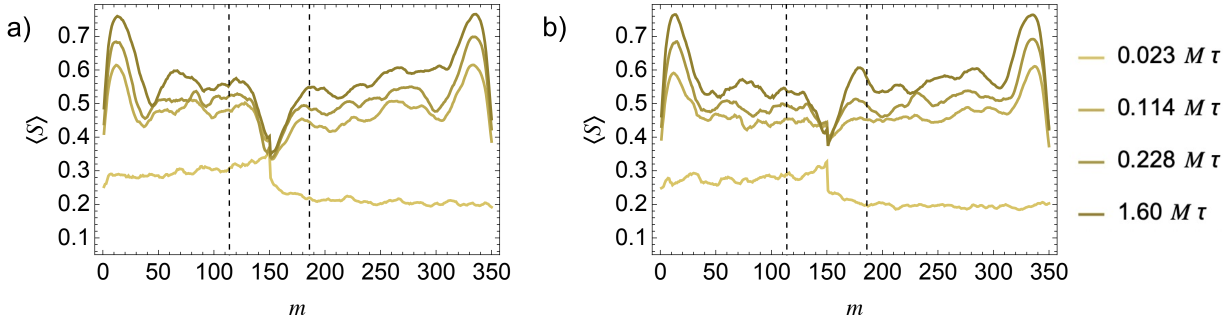

Figure S12: Average order parameter $\langle S \rangle$ vs. monomer index m for block copolymers in broad (a) and sharp (b) interfaces doped with $A_{150}B_{200}$ (n=12). The dashed lines mark $\pm N_e$ away from the junction point.

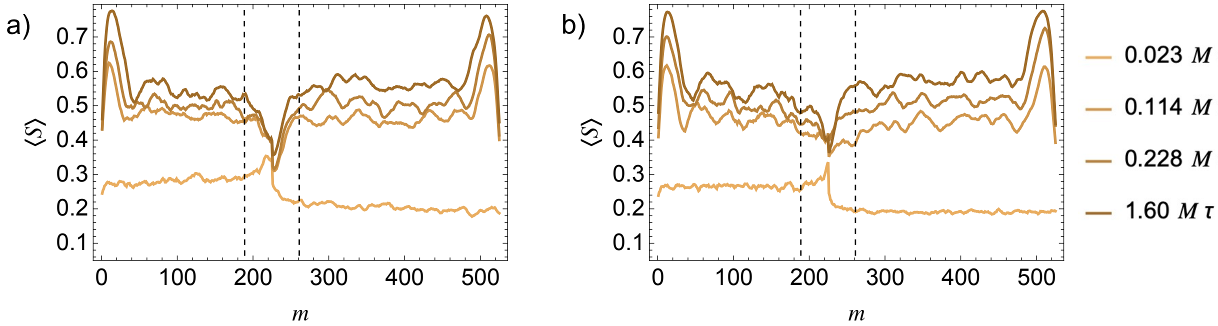

Figure S13: Average order parameter $\langle S \rangle$ vs. monomer index m for block copolymers in broad (a) and sharp (b) interfaces doped with $A_{225}B_{300}$ (n=12). The dashed lines mark $\pm N_e$ away from the junction point.

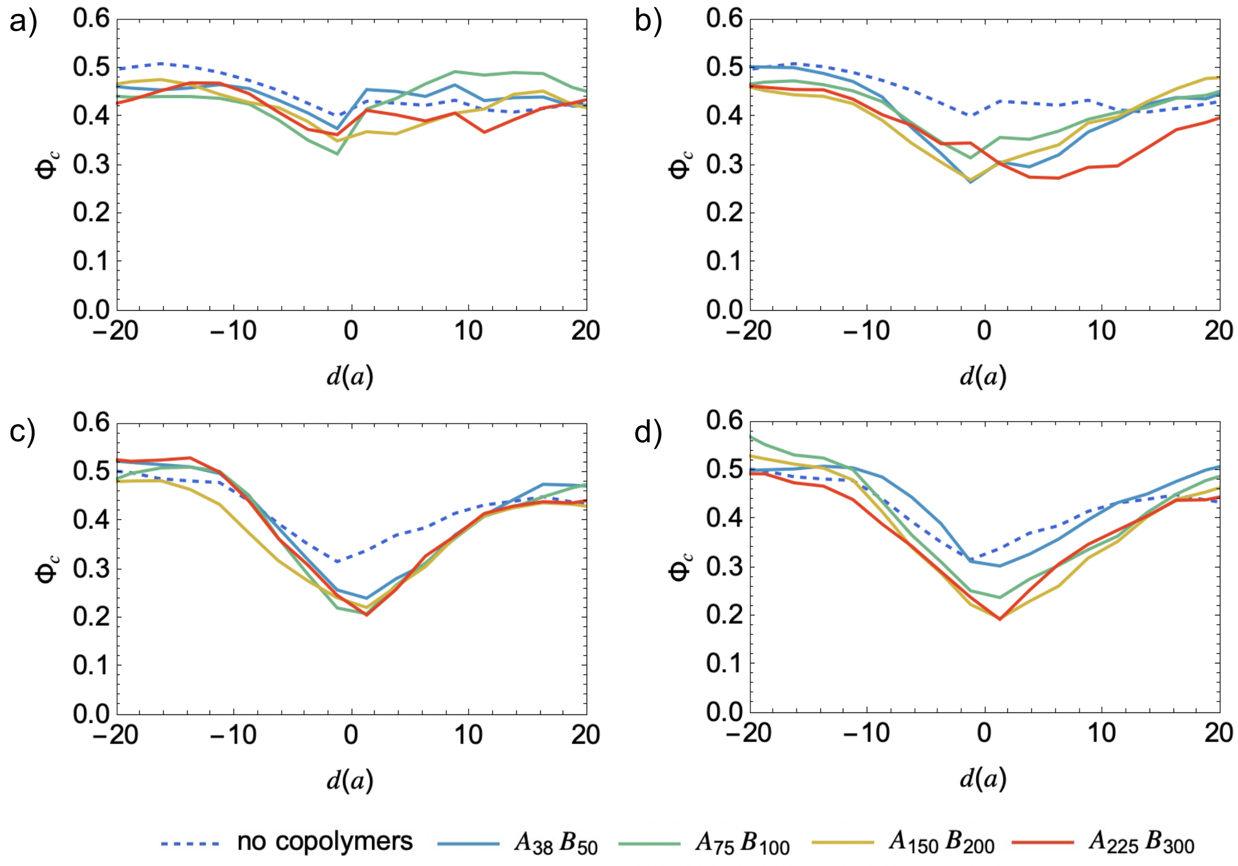

Figure S14: Crystallinity Φ_c of homopolymers alone vs. distance to the interface for sharp interfaces in (a) and (b), and broad interfaces in (c) and (d), under different copolymer loadings: $n = 12$ in (a, b) and $n = 24$ in (c, d), at the sharp (a, c) and broad (b, d) interfaces.

A crystal stem is defined as a contiguous segment of crystalline atoms along a single polymer chain. To account for minor defects, short amorphous interruptions containing two or fewer non-crystalline atoms between crystalline segments are still considered part of the same stem. Once all crystal stems are identified, their constituent atoms are assigned distances to the nearest interface. The stem lengths are then averaged vs. distance from the interface, yielding the spatial profile of average stem length N_c in Figure S15.

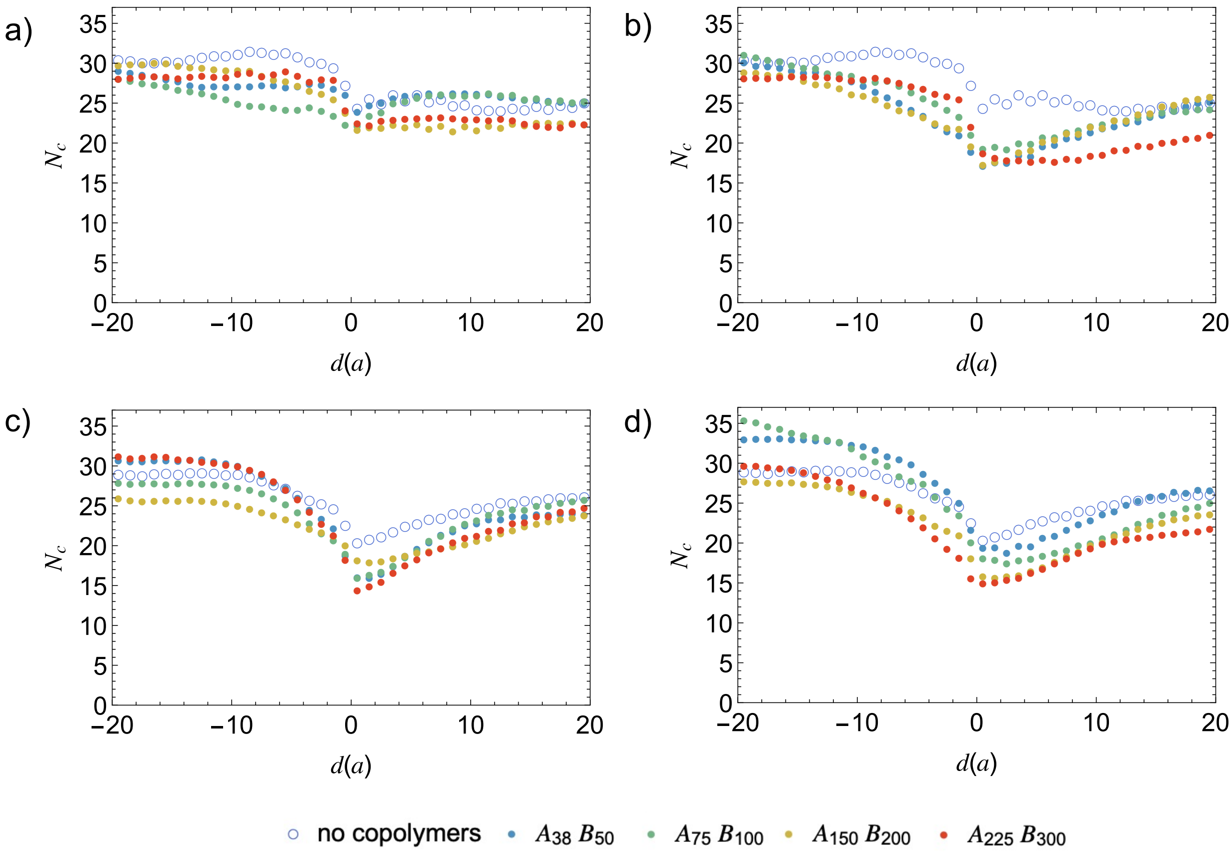

Figure S15: Crystal stem length N_c vs. distance to the interface for sharp interfaces in (a) and (b), and broad interfaces in (c) and (d), under different copolymer loadings: $n = 12$ in (a, b) and $n = 24$ in (c, d), at the sharp (a, c) and broad (b, d) interfaces.
